# Effect of different levels of feed restriction and fish oil fatty acid supplementation on fat deposition by using different techniques, plasma levels and mRNA expression of several adipokines in broiler breeder hens

**DOI:** 10.1371/journal.pone.0191121

**Published:** 2018-01-24

**Authors:** Namya Mellouk, Christelle Ramé, Maxime Marchand, Christophe Staub, Jean-Luc Touzé, Éric Venturi, Frédéric Mercerand, Angélique Travel, Pascal Chartrin, François Lecompte, Linlin Ma, Pascal Froment, Joëlle Dupont

**Affiliations:** 1 INRA UMR85 Physiologie de la Reproduction et des Comportements, Nouzilly, France; 2 CNRS UMR7247 Physiologie de la Reproduction et des Comportements, Nouzilly, France; 3 Université François Rabelais de Tours, Tours, France; 4 IFCE Nouzilly, France; 5 INRA—Unité Expérimentale du Pôle d’Expérimentation Avicole de Tours UEPEAT, Nouzilly, France; 6 INRA—Unité Expérimentale de Physiologie Animale de l’Orfrasière UEPAO 1297, Nouzilly, France; 7 ITAVI—Centre INRA Centre Val de Loire, Nouzilly, France; 8 INRA, Unité de Recherches Avicoles URA, Nouzilly, France; INIA, SPAIN

## Abstract

**Background:**

Reproductive hens are subjected to a restricted diet to limit the decline in fertility associated with change in body mass. However, endocrine and tissue responses to diet restriction need to be documented.

**Objective:**

We evaluated the effect of different levels of feed restriction, with or without fish oil supplementation, on metabolic parameters and adipokine levels in plasma and metabolic tissues of reproductive hens.

**Methods:**

We designed an *in vivo* protocol involving 4 groups of hens; RNS: restricted (Rt) unsupplemented, ANS: *ad libitum* (Ad, receiving an amount of feed 1.7 times greater than animals on the restricted diet) unsupplemented, RS: Rt supplemented, and AS: Ad supplemented. The fish oil supplement was used at 1% of the total diet composition.

**Results:**

Hens fed with the Rt diet had a significantly (*P* < 0.0001) lower growth than Ad hens, while the fish oil supplementation had no effect on these parameters. Furthermore, the bioelectrical impedance analysis (BIA) and the fat ultrasonographic examinations produced similar results to the other methods that required animals to be killed (carcass analysis and weight of adipose tissue). In addition, the Rt diet significantly (*P* < 0.05) decreased plasma levels of triglycerides, phospholipids, glucose and ADIPOQ, and fish oil supplementation decreased plasma levels of RARRES2. We also showed a positive correlation between insulin values and ADIPOQ or NAMPT or RARRES2 values, and a negative correlation of fat percentage to RARRES2 values. Moreover, the effects of the Rt diet and fish oil supplementation on the mRNA expression depended on the factors tested and the hen age.

**Conclusions:**

Rt diet and fish oil supplementation are able to modulate metabolic parameters and the expression of adipokines and their receptors in metabolic tissue.

## Introduction

Growth performance of breeder hens has increased spectacularly over the past several decades, mainly due to genetic progress and improvements in nutrition and management strategies. Unfortunately, this high growth rate has been accompanied by increased body fat deposition and high incidence of metabolic, skeletal and reproductive disorders [1]. Moreover, breeders have few non-invasive tools for estimating body fat composition in their animals which comply with ethical and welfare requirements. Consequently, this accentuates the difficulties in detecting metabolic diseases which occur in the case of overfeeding. Restriction of the consumption of *ad libitum* feed during rearing [2] and production is a common practice for reducing metabolic disorders and improving productivity [3, 4]. Indeed, improvements in metabolic deregulation and also in reproductive performance were shown in meat type chickens with restricted diets [5]. However, there is evidence that restricting feed intake leads to physiological stress, stereotypies, aggression, and other abnormal behaviour in poultry [6] [7]. Many studies comparing an *ad libitum* (Ad) diet *versus* a restricted (Rt) diet have shown the effects of Rt diet on welfare, growth, body composition and egg production [8] [9] [10]. Whilst much attention has been focused on the differences between restricted- and *ad libitum*-fed birds, less is documented on the endocrine response to long-term feed-restriction programmes. Supplementation with omega-3 polyunsaturated fatty acids (PUFAs) of marine origin was shown to improve zootechnical performance, such as body weight, laying rate, egg quality and fertility, in chickens [11]. Indeed, PUFAs are essential for development and growth in mammals. Omega-3 PUFAs mostly recognised for their beneficial effects on the metabolic and reproductive system are eicosapentanenoic acid (EPA) and docosahexaenoic acid (DHA) [12]. They can be added to the diet or synthetised from endogenous linoleic acid by the animals [13]. However, the effects of supplementation with these PUFAs on growth, fattening, metabolic parameters and fertility in Rt broiler breeder hens have not yet been fully investigated.

The metabolic changes due to nutritional status (Rt diet or omega 3 PUFAs supplementation) partly lead to variations in the regulation of metabolic hormonal profiles. In chickens, fasting reduces circulating 3,3′,5-triiodothyronine (T_3_), insulin, and insulin-like growth factor-I (IGF-I) levels [14], whereas plasma levels of glucocorticoid, insulin-like growth factor-II (IGF-II) [15], and growth hormone are increased [16]. A well-known effect of feed restriction in broiler breeder is the reduction of adiposity. The link between diet and body composition involves a regulation of the hormones secreted by adipose tissue, called adipokines. The most studied adipokine in mammals is leptin, but its existence has been long debated in birds [17] [18]. However, in recent years, many adipokines have been discovered in poultry, including adiponectin C1Q and collagen domain containing (ADIPOQ) [19], retinoic acid receptor responder 2 (RARRES2) [20] and nicotinamide phosphoribosyl transferase (NAMPT) [21] [22]. In chickens, ADIPOQ is involved in multiple processes, such as appetite, adipocyte differentiation, lipid metabolism and steroidogenesis [23–26]. Furthermore, NAMPT is mostly considered to be a myokine rather than an adipokine [21] and nothing has been described about chicken RARRES2. In hens and mice, dietary restriction decreases plasma leptin levels [27] [28] and the addition of PUFAs of marine origin leads to high concentrations of adiponectin [28]. Addition of EPA and DHA in mice and humans led to higher secretion of *ADIPOQ* [28] [29] [30]; only DHA enhanced *ADIPOQ* mRNA expression [29], whereas EPA increased *ADIPOQ* protein expression [30]. The effects of EPA and DHA appear to be mediated by the peroxisome proliferator-activated receptor gamma (*PPARG*). Furthermore, another study demonstrated that *in vivo* administration of EPA has a direct stimulatory effect on *NAMPT* gene expression and protein secretion in rat primary visceral adipose tissue cells [31]. The majority of studies on the incorporation of these fatty acids into the diets of chickens focus on their ability to produce meat or eggs for consumption that are richer in omega 3 [32], but less of the studies focus on the regulation of the metabolism of the animal itself. However, the pattern of expression of the recent adipokines in hens can be key indicators to help predict body composition and thus support management decisions in order to optimise production.

Thus, the present study was carried out to evaluate the impact of feeding conditions on metabolic parameters and to explore the plasma and tissue levels of adipokines, considered to be potential indicators of body mass in broiler breeder hens. Therefore, we performed an *in vivo* protocol to evaluate the effects of different levels of dietary restriction associated or not with fish oil supplementation on the body weight, fattening estimated using non-invasive (bioimpedance, ultrasonography and X-ray scanning analysis) and invasive techniques (carcass composition analysis and weight of abdominal fat) and plasma and tissue concentrations of various metabolic markers including adipokines.

## Materials and methods

### Ethical issues

All experimental procedures were performed in accordance with the French National Guidelines for the care and use of animals for research purposes (certificate of authorisation to experiment on living animals n°01607.02, Ministry of Agriculture and Fish Products, and favourable notice of ethics committee of Val de Loire N°19).

### Animals

Three hundred and twenty broiler breeder female chicks (Cobb 500) from Hendrix Genetics (Saint Laurent de la Plaine, France) were studied from day 1 to 39 weeks of age. Animals were distributed on the ground on the day of hatching, in homogeneous groups of 10 birds in 32 pens, each pen with an area of 3 m^2^. The animals were reared at ‘Pôle Expérimental Avicole de Tours’ (INRA, Nouzilly, France) according to the traditional conditions of breeding: 14 h of light per day on arrival, followed by a gradual decrease until reaching 8 h of light per day at the time of laying (week 21), and then a gradual increase until reaching 15 h of light per day at the end of the study (week 39). Animals were killed by electrical stunning and bled out as recommended by the ethical committee.

### Diets

From one to 28 days of age (week 4), female breeder chicks received a diet, called a starting diet, *ad libitum* (free access to food). At 28 days of age (week 4), animals were distributed into two treatment groups: the first group (n = 160 animals) received a restricted growing diet according to Hendrix Genetics recommendation; the second group (*ad libitum* group; n = 160 animals) received the same diet on a daily basis, but the amount was 1.7 times greater than in restricted animals. In order to adjust the amount of feed consumed by the animals, animals in two control pens (Rt) were weighed, compared and then feed adjusted weekly with respect to the theoretical curve provided by the supplier Cobb. From 63 days (week 9) to 273 days of age (week 39), the two treatment groups were each subdivided into two groups, one with fish oil supplementation and one without fish oil. The resulting four treatment groups were: group RNS (restricted unsupplemented); group ANS (*ad libitum* unsupplemented); group RS (restricted supplemented); group AS (*ad libitum* supplemented). During this period, these four groups of animals received three different diets (growing, before laying and during laying diets). The supplement was a protected encapsulated fish oil OMG750 provided by Kemin (Nantes, France) and was composed of refined fish oil (77%) and gelatin (capsule; 23%). The supplement was manually mixed into the diet at 1% of total diet (S1 Table and S2 Table and S1 Fig). Determination of the lipid composition of the fish oil supplement was performed by In Vivo Labs (Vannes, France) and is described in the S3 Table.

### Fatty acids profile in feed, egg yolk, adipose tissue, liver and muscle

The total lipids were extracted from the different diets (starting, growing, before laying and laying), egg yolk (week 25), adipose tissue, liver and muscle (week 39) after homogenization of the samples (n = 10 for each condition) with a chloroform/methanol mixture. Lipids were extracted gravimetrically into methanol:chloroform (1volume: 2 volume) according to Folch et al. [33]. The fatty acid composition was determined by gas chromatography (Autosystem; Perkin Elmer, St Quentin en Yvelines, France) after transmethylation of lipids (Morrisson and Smith, 1964).

### Determination of body weight, feed conversion and fattening

The fasted hens (n = 80 for each treatment) were weighed every 3 weeks using an automated balance from Grosseron (B146782- ENTRIS 8201i-1S, Coueron, France) with a precision of 0.1 g. Feed conversion was calculated as the ratio between the total feed intake during a fixed period (6 to 9 weeks, 9 to 18 weeks, 18 to 21 weeks or 23 to 39 weeks) and the total gain in body weight (6 to 9 weeks, 9 to 18 weeks and 18 to 21 weeks) or the total mass of eggs laid (23 to 39 weeks) during the same fixed period. The fat content of the chickens was estimated every 3 weeks by fat ultrasonographic examination (MyLab 30 Gold Vet,Hospimedi France, Saint-Crépin-Ibouvillers, France) and by the bioimpedance analysis (BIA, Quantum II–Body Composition Analyzer, RJL Systems, Clinton Township, Michigan, USA) (n = 80 animals for each treatment). The ultrasonographic examination determined (n = 80 animals per group the depth of adipose tissue on the back of animal and the BIA estimated percentage of fat in collaboration with INZO Laboratories (Argentan, France). The fat percentage of the whole carcass was estimated at 21 weeks (4 animals/group) and 39 weeks of age (4 animals/group). Briefly, animals were killed by cervical dislocation, feathers were removed and carcasses were frozen (−20°C). Each frozen carcass was first cut into small pieces with an electric band saw and ground with an electric tabletop meat grinder. Ground carcasses were freeze-dried and individually analysed for lipids, with no acidic treatment prior to lipid extraction by petroleum ether [34]. Fat content was estimated at 39 weeks of age by dissection and weighing the abdominal adipose tissue (n = 15 animals per group) and by scanning (n = 12 per group). Scanning was performed using an X-ray Computerized Tomography (CT) scanner (Siemens Somatom Definition AS). The X-ray source was set at 100 kV and 120 mA. Five hundred images were acquired every 0.6 mm, with a pitch of 0.45. The images were reconstructed using a reconstruction filter Safire I26. Phantoms of known fat tissue were used to calibrate the scanning parameters for adipose tissue measurement and phantoms were reconstructed under the same parameters as the animals. Analyses of all data were carried out using an Acquisition Sinogram Image Processing IDL's virtual machine (ASIPro VM, Siemens Medical Solutions).

### Plasma biochemical parameters

Blood samples were collected from the occipital sinus into heparin tubes at weeks 3, 9, 15, 18, 21, 27, 32 and 39 (8 animals /group). Plasma was recovered after centrifugation (5000 *g* for 10 min at 4°C) and then stored at -20°C until use. Plasma concentrations of glucose, cholesterol, triglycerides and phospholipids were determined by enzymatic assay using the GAGO-20 (Sigma Aldrich, Saint-Quentin Fallavier, France), the CHOD-PAP (Biolabo SAS, Maizy, France), the GPO method (Biolabo SAS, Maizy, France), and phospholipids assays (Biolabo SAS, Maizy, France), respectively. Plasma insulin concentration was determined by using a chicken-specific insulin ELISA kit (reference: abx512987-96, Holzel Diagnostika, Koln, Germany). The measurements were carried out according to the manufacturer's protocol.

### Adipokine assays

Plasma concentrations of adipokines (n = 8 for each condition) were obtained using chicken-specific kits: E12V0003 (sensitivity 1 ng/mL), E12A0125 (sensitivity 0.1 ng/mL) and E112C0104 (sensitivity 1 pg/mL) were used for NAMPT, ADIPOQ and RARRES2, respectively (Koln, Germany). The measurements were carried out according to the manufacturer's protocol with an intra-assay coefficient of variation < 6%. The absorbance was measured at 450 nm and then compared with reference values.

### Measurement of the surface area of the pectoral and oyster muscles after slaughter

The surface area of the major and minor pectoralis muscles (Pecto) and the oysters (located in the hollow on the dorsal side of the ilium bone) were measured at 39 weeks of age (n = 4 animals/group) by ultrasonography using a MyLab 30 Gold Vet ultrasound scanner (Hospimedi France, Saint-Crépin-Ibouvillers, France) equipped with two linear probes (Esaote L332 and L435, Hospimedi France, Saint-Crépin-Ibouvillers, France). The scanner operated at low frequency for the pectoral muscles (5 MHz) and a higher frequency for the oysters (15 MHz).

### mRNA expression of lipid metabolism factors, adipokines and their receptors in adipose tissue, liver and pectoralis muscle

Total RNA was extracted from the abdominal adipose tissue (AT abd), subcutaneous adipose tissue (AT sc), liver and Pecto from 39-week-old hens by homogenization in the TRIzol reagent using an Ultraturax, according to the manufacturer's recommendations (Invitrogen by Life Technologies, Villebon sur Yvette, France). The cDNA was generated by reverse transcription (RT) of total RNA (1 μg) in a mixture comprising 0.5 mM of each deoxyribonucleotide triphosphate (dATP, dGTP, dCTP and DTTP), 2 M of RT buffer, 15 μg/μL of oligodT, 0.125 U of ribonuclease inhibitor, and 0.05 U of Moloney murine leukemia virus reverse transcriptase (MMLV) for one hour at 37°C. Real-time PCR was performed using the MyiQ Cycle device (Bio-Rad, Marnes-la-Coquette, France), in a mixture containing SYBR Green Supermix 1X reagent (Bio-Rad, Marnes la Coquette, France), 250 nM specific primers (Invitrogen by Life Technologies, Villebon sur Yvette, France) (S4 Table) and 5 μL of cDNA (diluted five-fold) for a total volume of 20 μL The samples were duplicated on the same plate and the following PCR procedure used: after an incubation of 2 min at 50°C and a denaturation step of 10 min at 95°C, samples were subjected to 40 cycles (30 s at 95°C, 30 s at 60°C and 30 s at 72°C). The levels of expression of messenger RNA were standardised to three reference genes (*RPL15*, *EF1 and β actin*). For each gene, the relative abundance of transcription was determined by the calculation of e^-ct^ The relative expression of the gene of interest was then related to the relative expression of the geometric mean of the three reference genes.

### Statistical analysis

SAS software (version 9.3) was used for all analyses. A two-way ANOVA test in repeated measurements was used to compare the mean values for body weight, feed intake, fat content and plasma concentrations of biochemical parameters between different treatment groups from the beginning of the experiment to week 39.

The model used was:

$$Y_{ijkl}=\mu +Diet_{i}+Supp_{j}{}^{}+Wk_{k}+Diet_{i}\;*\;Supp_{j}+Diet_{i}\;*\;Wk_{k}+Diet_{i}\;*\;Wk_{k}+e_{ijkl}$$

where Y_ijkl_ is the dependent variable (body weight, feed intake, fat [ultrasound and BIA] and plasma biochemical parameters), μ is the overall mean, Diet_i_ is the fixed effect of diet i (I = Rt, Ad), Supp_j_ is the fixed effect of fish oil j (j = supplemented, unsupplemented), Wk_k_ is the fixed effect of week k (k = 1, 2), Diet_i_ * Supp_j_ is the interaction between Diet_i_ and Supp_j_, Diet_i_ * Wk_k_ is the interaction between Diet_i_ and Wk_k_, and e_ijkl_ is the residual error.

Then, for plasma hormone concentrations, we applied a similar model for different periods (week 3 to 9, week 9 to 18, week 18 to 21 and week 21 to 39) without week effect.

The model used was:

$$Y_{ijkl}=\mu +Diet_{i}+Supp_{j}{}^{}+Diet_{i}\;*\;Supp_{j}+e_{ijk}$$

where Y_ijkl_ is the dependent variable (triglyceride, phospholipid, cholesterol, glucose, insulin, ADIPOQ, NAMPT and RARRES2), μ is the overall mean, Diet_i_ is the fixed effect of diet i (I = Rt, Ad), Supp_j_ is the fixed effect of fish oil j (j = supplemented, unsupplemented), Diet_i_ * Supp_j_ is the interaction between Diet_i_ and Supp_j_, and e_ijk_ is the residual error.

At week 9, we only analysed the effect of the Rt diet, as animals were separated into only two groups (Rt and Ad animals) after blood sampling. For parameters measured only at a specific time point (feed conversion, fat content by carcass analysis and scanner, weight of abdominal adipose tissue and mRNA expression), we used a two way ANOVA. The results are represented as mean ± SEM, with *P* < 0.05 being considered significant.

A Pearson test was used for the correlation analyses between the different parameters and the correlation coefficient is noted as “r”, with *P* < 0.05 being considered significant.

## Results

### Effect of restricted diet and fish oil supplementation on fatty acid composition in egg yolk at 39 weeks

As shown in Table 1, the intake of fish oil resulted in a change in the fatty acid composition of egg yolk of hens. The fish oil supplementation (1% of total feed) used is sufficient to cause a significant increase of n-3 fatty acids (*P* <0.0001), including EPA (C20:5 n-3, *P* <0.0001), docosapentaenoic acid (DPA, C22:5 n-3, *P* <0.0001) and DHA (C22:6 n-3, *P* <0.0001), while we observed a decrease in alpha-linolenic acid (ALA, C18:3, *P* = 0.004). The fish oil supplementation also significantly decreased n-6 fatty acids (*P* = 0.05), such as arachidonic acid (AA, C20:4 n-6, *P* = 0.05) and docosapentaenoic acid (Osbond acid, C22:4 n-6, *P* <0.0001). These effects induced a decrease in the n-6/n-3 ratio (*P* < 0.0001). However, no effect of the diet and on variation of LA (C18:2) incorporation were noted between the different groups (Table 1).

**Table 1:** Proportion of fatty acids in egg yolk at 39 weeks (n = 10 for each condition).

		Ad		Rt		P-value	
	Ad	Supp	Ctrl	Supp	Diet	Supp	Diet*Supp
C18:2	20.65 ± 0.47	20.37 ± 0.34	21.67 ± 0.47	20.82 ± 0.26	0.07	0.15	0.46
C18:3	1.08 ± 0.05	0.95 ± 0.02	1.10 ± 0.05	0.98 ± 0.03	0.43	**0.004**	0.89
C20:4 n-6	1.47 ± 0.13	1.32 ± 0.06	1.60 ± 0.13	1.33 ± 0.07	0.48	**0.05**	0.56
C20:5 n-3	0.06 ± 0.01	0.11 ± 0.01	0.06 ± 0.02	0.13 ± 0.01	0.52	**<0.0001**	0.52
C22:4 n-6	0.19 ± 0.03	0.09 ± 0.01	0.19 ± 0.03	0.08 ± 0.01	0.93	**<0.0001**	0.97
C22:5 n-3	0.29 ± 0.02	0.38 ± 0.02	0.27 ± 0.02	0.41 ± 0.03	0.96	**<0.0001**	0.35
C22:6 n-3	1.33 ± 0.11	2.19 ± 0.11	1.33 ± 0.11	2.11 ± 0.11	0.73	**<0.0001**	0.74
n-6	22.32 ± 0.53	21.79 ± 0.34	23.47 ± 0.54	22.24 ± 0.29	0.07	**0.05**	0.43
n-3	2.78 ± 0.10	3.64 ± 0.12	2.78 ± 0.13	3.64 ± 0.12	0.97	**<0.0001**	0.98
n-6/n-3	8.09 ± 0.27	6.06 ± 0.26	8.58 ±0.43	6.15 ±0.20	0.33	**<0.0001**	0.51

Results are presented as lsmeans ± SEM. P values of the effects of diet, supplementation and the interaction between diet and supplementation were considered as significant if *P* ≤ 0.05. Ad: *ad libitum*, Rt: restricted, Supp: supplemented, Ctrl: control unsupplemented

### Effect of restricted diet and fish oil supplementation on fatty acid composition in liver at 39 weeks

In liver, the incorporation of fatty acids was affected by fish oil supplementation and also by the diet. Indeed, we observed a decrease in n-6 fatty acids (*P* = 0.0004), mainly linoleic acid (LA, C18:2, *P* = 0.0001), AA (C20:4 n-6, *P* < 0.0001) and Osbond acid (C22:4 n-6, *P* = 0.001). No effect of the fish oil supplementation was observed on total n-3 fatty acids, even if it decreased ALA (C18:3, *P* = 0.0002) and increased EPA (C20:5 n-3, *P* = 0.005). The Rt diet had a different effect on n-3 and n-6 fatty acids compared to fish oil supplementation. The Rt diet increased the incorporation of total n-3 and n-6 fatty acids, while it decreased the n-6/n-3 ratio. This effect can be explained by an increase in EPA (C20:5 n-3, *P* = 0.0002), DPA (C22:5 n-3, *P* < 0.0001), DHA (C22:6 n-3, *P* < 0.0001), LA (C18:2, *P* = 0.01), AA (C20:4 n-6, *P* = 0.01) and Osbond acids (C22:4 n-6, *P* < 0.0001) (Table 2).

**Table 2:** Proportion of fatty acids in liver at 39 weeks (n = 10 for each condition).

		Ad		Rt		P-value	
	Ctrl	Supp	Ctrl	Supp	Diet	Supp	Diet*Supp
C18:2	16.55 ± 0.95	12.47 ± 0.51	18.18 ± 0.69	14.96 ± 1.10	**0.01**	**0.0001**	0.61
C18:3	0.63 ± 0.05	0.44 ± 0.04	0.58 ± 0.03	0.44 ± 0.04	0.43	**0.0002**	0.48
C20:4 n-6	3.64 ± 0.66	1.44 ± 0.20	6.50 ± 0.82	5.24 ± 0.70	**0.01**	**<0.0001**	0.46
C20:5 n-3	0.02 ± 0.004	0.04 ± 0.01	0.06 ± 0.02	0.13 ± 0.02	**0.0002**	**0.005**	0.17
C22:4 n-6	0.38 ± 0.09	0.10 ± 0.01	0.75 ± 0.10	0.50 ± 0.06	**<0.0001**	**0.001**	0.08
C22:5 n-3	0.17 ± 0.04	0.08 ± 0.01	0.35 ± 0.05	0.34 ± 0.05	**<0.0001**	0.26	0.30
C22:6 n-3	2.14 ± 0.36	1.49 ± 0.21	2.84 ± 0.23	4.50 ± 0.64	**<0.0001**	0.21	0.006
n-6	20.58 ± 1.57	14.01 ± 0.69	25.44 ± 1.49	20.70 ± 1.80	**0.0003**	**0.0004**	0.53
n-3	2.97 ± 0.41	2.06 ± 0.26	3.84 ± 0.28	5.42 ± 0.73	**<0.0001**	0.46	0.01
n-6/n-3	7.58 ± 0.58	7.25 ± 0.45	6.79 ±0.40	4.20 ±0.37	**0.0002**	**0.003**	0.01

Results are presented as lsmeans ± SEM. P values of the effects of diet, supplementation and the interaction between diet and supplementation were considered as significant if *P* ≤ 0.05. Ad: *ad libitum*, Rt: restricted, Supp: supplemented, Ctrl: control unsupplemented

### Effect of restricted diet and fish oil supplementation on fatty acid composition in adipose tissue at 39 weeks

Similar to the liver, the incorporation of fatty acids in adipose tissue is dependent on the diet quantity and its fatty acid components. Fish oil supplementation decreased the incorporation of LA (C18:2, *P* = 0.004) and ALA (C18:3, *P* < 0.0001), whereas it increased the incorporation of EPA (C20:5 n-3, *P* = 0.03) and DHA (C22:6 n-3, *P* = 0.05). These effects were associated with a decrease in total n-3 (*P* = 0.0002) and n-6 (*P* = 0.004) fatty acids and an increase in the n-6/ n-3 ratio (*P* = 0.005). In addition, the Rt diet improved the incorporation of LA (C18:2, *P* = 0.005) and AA (C20:4 n-6, *P* = 0.01), leading to an increase of total n-6 fatty acid (*P* = 0.005). However, the Rt diet decreased the DPA (C22:5 n-3, *P* = 0.01) incorporation, which was insufficient to affect the total n-3 fatty acid content as well as the n-6/n-3 ratio (Table 3).

**Table 3:** Proportion of fatty acids in adipose tissue at 39 weeks (n = 10 for each condition).

		Ad		Rt		P-value	
	Ctrl	Supp	Ctrl	Supp	Diet	Supp	Diet*Supp
C18:2	32.54 ± 1.34	26.67 ± 0.94	33.29 ± 1.25	32.48 ± 0.83	**0.005**	**0.004**	0.02
C18:3	2.63 ± 0.13	1.86 ± 0.11	2.52 ± 0.09	2.30 ± 0.10	0.12	**<0.0001**	0.01
C20:4 n-6	0.09 ± 0.01	0.06 ± 0.01	0.09 ± 0.01	0.11 ± 0.01	**0.01**	0.83	0.01
C20:5 n-3	0.02 ± 0.004	0.03 ± 0.004	± 0.006	0.04 ± 0.01	0.30	**0.03**	0.58
C22:4 n-6	0.03 ± 0.003	0.02 ± 0.005	0.02 ± 0.003	0.03 ± 0.003	0.88	0.88	0.21
C22:5 n-3	0.02 ± 0.004	0.02 ± 0.004	0.02 ± 0.004	0.042 ± 0.007	**0.01**	0.15	0.15
C22:6 n-3	0.03 ± 0.005	0.04 ± 0.009	0.03 ± 0.005	0.07 ± 0.02	0.17	**0.05**	0.26
n-6	32.66 ± 1.34	26.771 ± 0.95	33.41 ± 1.25	32.63 ± 0.83	**0.005**	**0.004**	0.02
n-3	2.70 ± 0.13	1.96 ± 0.11	2.61 ± 0.09	2.46 ± 0.09	0.05	**0.0002**	0.008
n-6/n-3	12.14 ± 0.26	13.88 ± 0.55	12.78 ± 0.17	13.32 ± 0.43	0.90	**0.005**	0.12

Results are presented as lsmeans ± SEM. P values of the effects of diet, supplementation and the interaction between diet and supplementation were considered as significant if *P* ≤ 0.05. Ad: *ad libitum*, Rt: restricted, Supp: supplemented, Ctrl: control unsupplemented

### Effect of restricted diet and fish oil supplementation on fatty acid composition in muscle tissue at 39 weeks

As shown in Table 4, we found that animals fed with a diet supplemented with fish oil had lower percentage of n-6 fatty acids (*P* < 0.0001) and higher n-3 fatty acids (*P* = 0.0004), leading to a lower n-6/n-3 ratio (*P* < 0.0001) in thoracic limb muscle as compared to animals fed with an unsupplemented diet. These effect were mostly due to a lower incorporation of LA (C18:2, *P* < 0.0001), AA (C20:4 n-6, *P* = 0.01) and Osbond acid (C22:4 n-6, *P* < 0.0001), as well as higher incorporation of EPA (C20: n-3, *P* < 0.0001), DPA (C22:5 n-3, *P* = 0.0008) and DHA (C22:6 n-3, *P* < 0.0001). However, the decreasing effect on ALA (C18:3) was not sufficient to reverse the effect on n-3 fatty acids. Furthermore, we showed an effect of diet on the percentage of LA (C18:2, *P* = 0.0007), AA (C20:4 n-6, *P* = 0.006) and Osbond acid (C22:4 n-6, *P* = 0.01), leading to a higher percentage of n-6 fatty acids (*P* < 0.0001). We also observed that the Rt diet increased the percentage of DPA (C22:5 n-3, *P* = 0.03) in thoracic limb muscle, but it did not affect the percentage of total n-3 fatty acids and the n-6/n-3 ratio (Table 4).

**Table 4:** Proportion of fatty acids in thoracic limb at 39 weeks (n = 10 for each condition).

		Ad		Rt		P-value	
	Ctrl	Supp	Ctrl	Supp	Diet	Supp	Diet*Supp
C18:2	26.08 ± 0.57	22.25 ± 0.33	27.58 ± 0.76	25.54 ± 0.80	**0.0007**	**<0.0001**	0.17
C18:3	1.72 ± 0.11	1.26 ± 0.07	1.70 ± 0.10	1.40 ± 0.10	0.56	**0.0004**	0.42
C20:4 n-6	3.49 ± 0.17	2.26 ± 0.23	4.28 ± 0.58	3.65 ± 0.38	**0.006**	**0.01**	0.44
C20:5 n-3	0.08 ± 0.01	0.29 ± 0.03	0.07 ± 0.01	0.33 ± 0.05	0.55	**<0.0001**	0.36
C22:4 n-6	0.21 ± 0.01	0.07 ± 0.01	0.27 ± 0.04	0.14 ± 0.01	**0.01**	**<0.0001**	0.77
C22:5 n-3	0.46 ± 0.03	0.60 ± 0.06	0.56 ± 0.07	0.77 ± 0.08	**0.03**	**0.008**	0.59
C22:6 n-3	1.11 ± 0.06	1.71 ± 0.22	1.00 ± 0.13	2.17 ± 0.28	0.37	**<0.0001**	0.15
n-6	29.80 ± 0.49	24.59 ± 0.42	32.14 ± 0.72	29.34 ± 0.80	**<0.0001**	**<0.0001**	0.06
n-3	3.39 ± 0.08	3.88 ± 0.27	3.35 ± 0.17	4.68 ± 0.33	0.11	**0.0004**	0.07
n-6/n-3	8.79 ± 0.15	6.58 ± 0.41	9.73 ±0.38	6.61 ± 0.60	0.25	**<0.0001**	0.27

Results are presented as lsmeans ± SEM. P values of the effects of diet, supplementation and the interaction between diet and supplementation were considered as significant if *P* ≤ 0.05. Ad: *ad libitum*, Rt: restricted, Supp: supplemented, Ctrl: control unsupplemented

We found a similar effect of fish oil supplementation on LA, ALA, EPA, Osbond acid, DHA and total n-6 fatty acids in the pelvic limb muscle, but it had no effect on AA, DPA, total n-3 fatty acids and the n-6/n-3 ratio. Concerning the effect of the Rt diet in pelvic limbs, it still affected LA and n-6 fatty acids, it decreased the percentage of EPA and had no effect on the other fatty acids or the n-6/n-3 ratio (Table 5).

**Table 5:** Proportion of fatty acids in pelvic limb at 39 weeks (n = 10 for each condition).

		Ad		Rt		P-value	
	Ctrl	Supp	Ctrl	Supp	Diet	Supp	Diet*Supp
C18:2	27.76 ± 0.61	24.20 ± 0.67	29.43 ± 1.14	28.54 ± 0.88	**0.002**	**0.02**	0.15
C18:3	2.03 ± 0.15	1.50 ± 0.11	2.02 ± 0.09	1.76 ±0.01	0.34	**0.002**	0.29
C20:4 n-6	1.73 ± 0.26	1.51 ± 0.11	1.90 ± 0.34	1.71 ± 0.01	0.52	0.49	0.94
C20:5 n-3	0.12 ± 0.03	0.24 ± 0.04	0.07 ± 0.02	0.14 ± 0.002	**0.02**	**0.005**	0.39
C22:4 n-6	0.18 ± 0.04	0.08 ± 0.02	0.14 ± 0.03	0.10 ± 0.002	0.72	**0.01**	0.30
C22:5 n-3	0.15 ± 0.02	0.23 ± 0.01	0.22 ± 0.03	0.26 ± 0.004	0.21	0.10	0.67
C22:6 n-3	0.41 ± 0.08	0.59 ± 0.04	0.45 ± 0.07	0.72 ± 0.03	0.41	**0.03**	0.67
n-6	29.68 ± 0.83	25.80 ± 0.70	31.48 ± 1.24	30.36 ± 0.87	**0.002**	**0.01**	0.17
n-3	2.73 ± 0.22	2.58 ± 0.16	2.78 ± 0.13	2.89 ± 0.04	0.33	0.91	0.47
n-6/n-3	11.39 ± 0.85	10.31 ±0.68	11.37 ± 0.34	10.76 ± 0.15	0.73	0.19	0.71

Results are presented as lsmeans ± SEM. P values of the effects of diet, supplementation and the interaction between diet and supplementation were considered as significant if *P* ≤ 0.05. Ad: *ad libitum*, Rt: restricted, Supp: supplemented, Ctrl: control unsupplemented

### Effect of restricted diet and fish oil supplementation on body weight, fattening, feed conversion from 3 to 39 weeks and muscle surface area at slaughter

**Fig 1:**
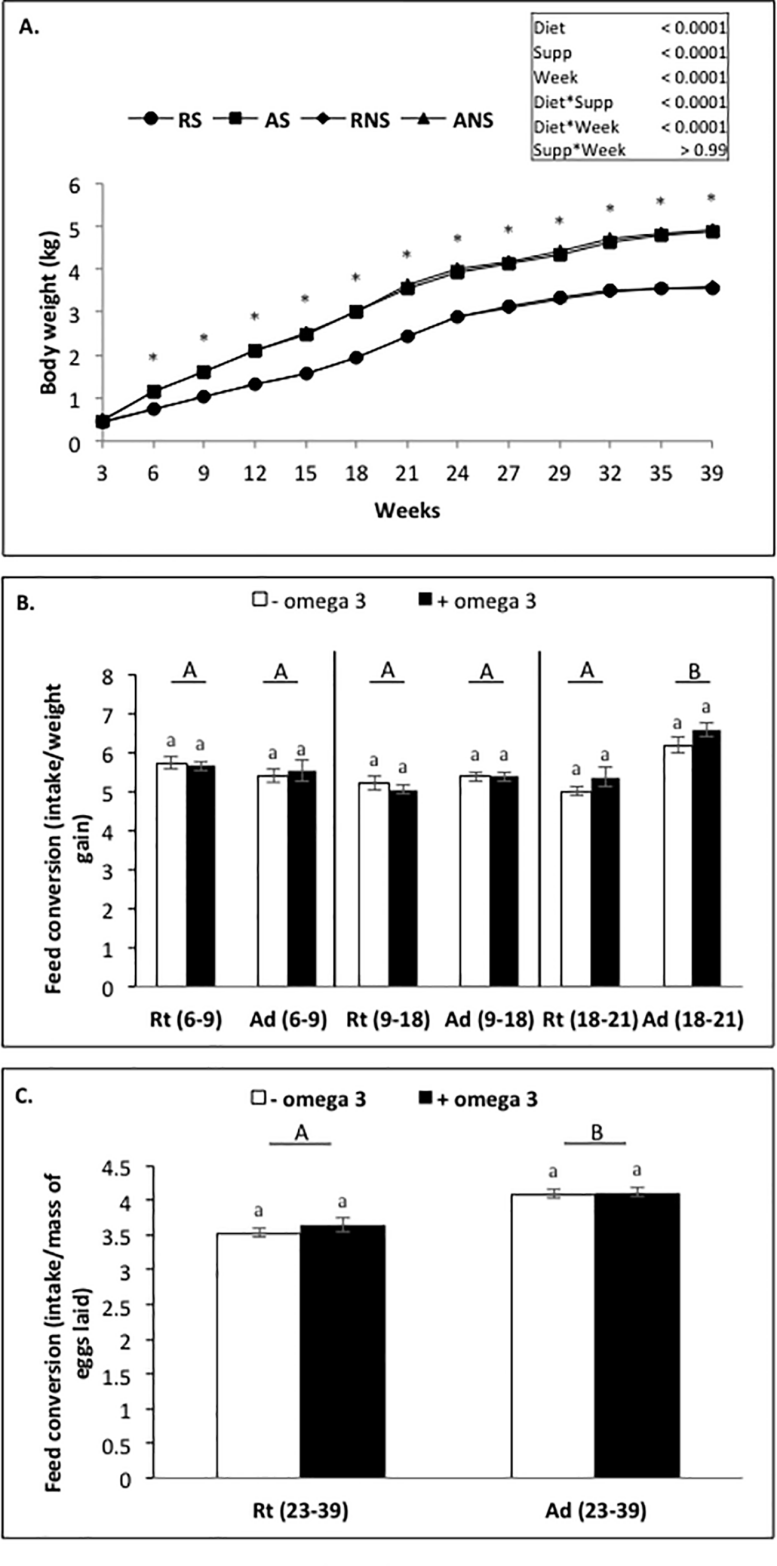
**Variation of body weight (A) and feed conversion (B and C) in broiler hens fed *ad libitum* or with a restricted diet, with or without fish oil supplementation.** RS: animals fed with restricted and supplemented diet (n = 80), AS: animals fed *ad libitum* with supplemented diet (n = 80), RNS: animals fed with restricted and unsupplemented diet (n = 80), ANS: animals fed *ad libitum* with unsupplemented diet (n = 80). Results are presented as lsmeans ± s.e.m. *P < 0.05 (diet effect) and *P* < 0.05 (fish oil supplementation effect). Different letters indicate significant differences. Capital letters indicate a significant effect of the diet and lower case letters indicate a significant effect of fish oil supplementation.

**Fig 2:**
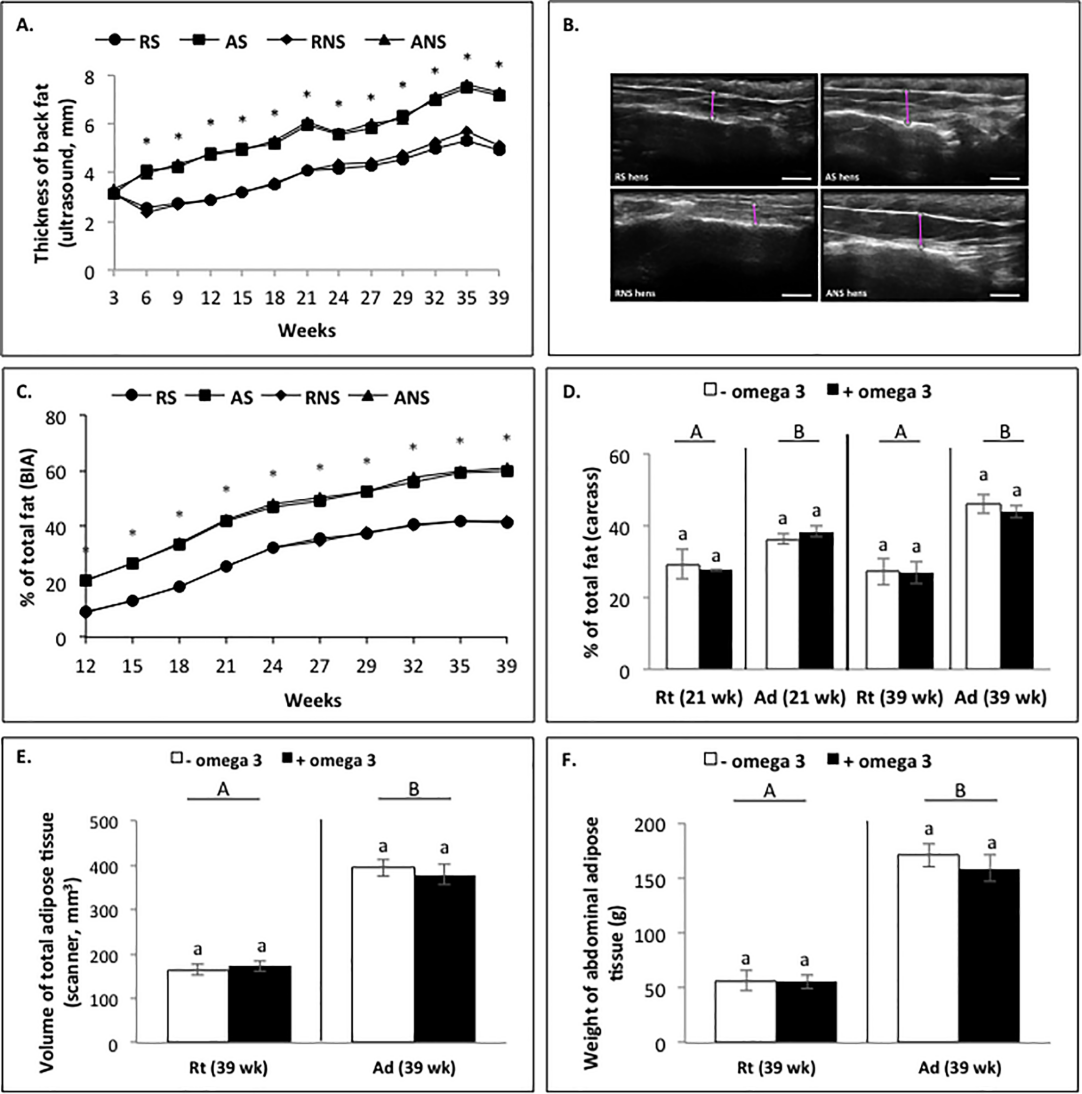
Variation of the fattening of broiler hens fed *ad libitum* or with a restricted diet, with or without fish oil supplementation. The fattening was assessed by ultrasound examinations (A, F, n = 80 animals/group) every three weeks from week 3 to week 39, BIA (B, n = 80 animals/group) every three weeks from week 12 to week 39, carcass analysis (C) at week 21 (n = 4 animals/group) and week 39 (n = 4 animals/group), weighing of abdominal adipose tissue (D, n = 15 animals/group) and by X-ray computerised tomography scanner (E, n = 12 animals/group) at week 39. RS: animals fed with restricted and supplemented diet, AS: animals fed *ad libitum* with supplemented diet, RNS: animals fed with restricted and unsupplemented diet, ANS: animals fed *ad libitum* with unsupplemented diet, Rt: animals fed with restricted diet, Ad: animals fed with *ad libitum* diet, wk: weeks. Results are presented as lsmeans ± s.e.m. *P < 0.05 (diet effect) and°*P* < 0.05 (fish oil supplementation effect). Different letters indicate significant differences. Capital letters indicate a significant effect of the diet and lower case letters indicate a significant effect of fish oil supplementation.

From week 3 to week 39, we observed a weak effect (*P* < 0.0001) on body weight and fattening (ultrasound, BIA) that increased gradually throughout the study. We also observed an effect of the diet (*P* < 0.0001) on body weight and fattening (ultrasound, BIA), as well as an effect of fish oil supplementation (*P* < 0.0001) (only fattening by ultrasound) (Table 6). The effect of diet on body weight and fattening was reported for each period according to the different feed (growing: week 6 to 9 and week 9 to 18, *P* < 0.0001; before laying: week 18 to 21, *P* < 0.0001; and laying: week 24 to 39, *P* < 0.0001; Table 6). More precisely, Rt animals reduced body weight gain as compared to Ad animals from 6 (0.77 ± 0.01 kg *vs* 1.15 ± 0.008 kg, respectively; *P* < 0.0001) to 39 weeks (3.56 ± 0.05 kg *vs* 4.89 ± 0.05 kg, respectively; *P* < 0.0001) (Fig 1A). We also observed a decrease in feed conversion in Rt animals compared to Ad animals from week 18 (week 18 to 21: 5.19 ± 1.46 *vs* 6.36 ± 1.43, respectively; *P* < 0.0001, and week 23 to 39: 3.60 ± 0.63 *vs* 4.12 ± 0.52, respectively; *P* < 0.0001) (Fig 1B and 1C). A similar negative effect of the Rt diet was noted for fattening from the second ultrasound measurement (week 6: 2.5 ± 0.04 mm *vs* 4.0 ± 0.1 mm, respectively; *P* < 0.001) (Fig 2A and 2B). Regarding to the different time periods, fish oil supplementation during the growing period increased only the body weight (week 9 to 18: 1.77 ± 0.03 kg *vs* 2.07 ± 0.03 kg, *P* < 0.0001) and, during the laying period, decreased only the fattening (ultrasound) (week 24 to 39: 5.76 ± 0.07 mm *vs* 5.6 ± 0.06 mm; Table 6). However, when we tested the effect of the fish oil supplementation week by week, no significant effect was observed on body weight or fattening anymore (Figs 1A, 2A and 2B).

**Table 6:** Effect of week, diet and fish oil supplementation on body weight and fattening (ultrasound or BIA) of hens fed *ad libitum* (Ad) or restricted (Rt) either with (Supp) or without (Crtl) fish oil supplementation (n = 80 for each condition).

			Body weight (kg)	Fat (ultrasound, mm)	Fat (BIA, %)
0 to 39	Ad	Crtl	2.31 ± 0.04 ᵃ	4.92 ± 0.04 ᵃ	44.0 ± 5 ᵃ
		Supp	3.70 ± 0.04 ᵇ	5.94 ± 0.05 ᵇ	43.3 ± 5 ᵃ
	Rt	Crtl	2.07 ± 0.03 ᶜ	3.71 ± 0.04 ᶜ	28.5 ± 5 ᵇ
		Supp	2.65 ± 0.03 ᵈ	4.11 ± 0.04 ᵈ	28.3 ± 5 ᵇ
		Diet	**< 0.0001**	**< 0.0001**	**< 0.0001**
		Supp	**< 0.0001**	**< 0.0001**	0.110
		Week	**< 0.0001**	**< 0.0001**	**< 0.0001**
	*P*	Diet*Supp	**< 0.0001**	**< 0.0001**	0.14
		Diet*Week	**< 0.0001**	**< 0.0001**	**< 0.0001**
		Supp*Week	> 0.99	0.97	0.99
6 to 9	Ad	Crtl	1.37 ± 0.02 ᵃ	4.12 ± 0.05 ᵃ	
	Rt	Crtl	0.88 ± 0.008 ᵇ	2.58 ± 0.03 ᵇ	
	*P*	Diet	**< 0.0001**	**< 0.0001**	
9 to 18	Ad	Crtl	2.17 ± 0.03 ᵃ	4.82 ± 0.06 ᵃ	26.9 ± 4 ᵃ
		Supp	2.52 ± 0.03 ᵃ	4.78 ± 0.06 ᵃ	26.7 ± 4 ᵃ
	Rt	Crtl	1.37 ± 0.02 ᵇ	3.08 ± 0.05 ᵇ	13.3 ± 3 ᵇ
		Supp	1.61 ± 0.02 ᵇ	3.07 ± 0.04 ᵇ	13.3 ± 3 ᵇ
	*P*	Diet	**< 0.0001**	**< 0.0001**	**< 0.0001**
		Supp	**< 0.0001**	0.57	0.76
		Diet*Supp	**0.02**	0.80	0.72
18 to 21	Ad	Crtl	3.30 ± 0.04 ᵃ	5.69 ± 0.09 ᵃ	38.0 ± 5 ᵃ
		Supp	3.27 ± 0.04 ᵃ	5.56 ± 0.09 ᵃ	37.5 ± 5 ᵃ
	Rt	Crtl	2.18 ± 0.03 ᵇ	3.83 ± 0.08 ᵇ	21.9 ± 5 ᵇ
		Supp	2.18 ± 0.03 ᵇ	3.78 ± 0.07 ᵇ	21.7 ± 4 ᵇ
	*P*	Diet	**< 0.0001**	**< 0.0001**	**< 0.0001**
		Supp	0.68	0.28	0.48
		Diet*Supp	0.68	0.61	0.68
24 to 39	Ad	Crtl	4.50 ± 0.03 ᵃ	6.61 ± 0.07 ᵃ	54.6 ± 4 ᵃ
		Supp	4.43 ± 0.03 ᵃ	6.52 ± 0.07 ᵇ	53.8 ± 4 ᵃ
	Rt	Crtl	3.31 ± 0.02 ᵇ	4.90 ± 0.06 ᶜ	38.0 ± 4 ᵇ
		Supp	3.32 ± 0.02 ᵇ	4.68 ± 0.05 ᶜ	37.9 ± 3 ᵇ
	*P*	Diet	**< 0.0001**	**< 0.0001**	**< 0.0001**
		Supp	0.24	**0.02**	0.28
		Diet*Supp	0.15	0.26	31

Results are presented as lsmeans ± SEM. P values of the effects of diet, supplementation, week and the interaction between diet and supplementation, diet and week, and supplementation and week were considered as significant if *P* ≤ 0.05. Different individual letters in superscript (a,b,c and d) indicate significant differences.

**Fig 3:**
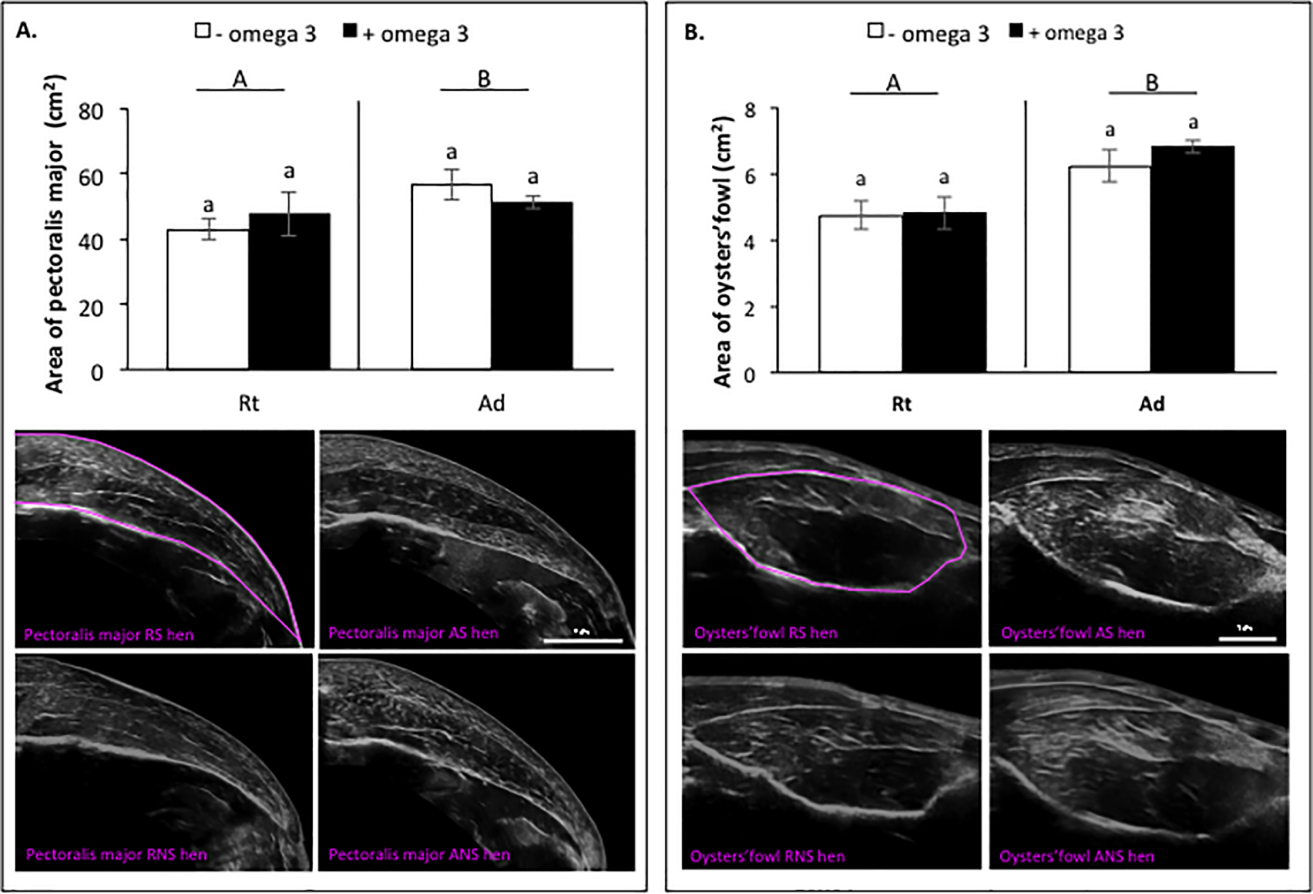
**Surface area of pectoralis major muscle (A) and oyster muscle (B) in broiler hens fed *ad libitum* or with a restricted diet, with or without fish oil supplementation at 39 weeks.** The surface area of muscles was assessed by ultrasound examination. RS: animals fed with restricted and supplemented diet, AS: animals fed *ad libitum* with supplemented diet, RNS: animals fed with restricted and unsupplemented diet, ANS: animals fed *ad libitum* with unsupplemented diet, Rt: animals fed with restricted diet, Ad: animals fed *ad libitum*. Results are presented as lsmeans ± s.e.m. *P < 0.05 (diet effect) and°*P* < 0.05 (fish oil supplementation effect). Different letters indicate significant differences. Capital letters indicate a significant effect of the diet and lower case letters indicate a significant effect of the fish oil supplementation.

In addition, we showed a positive correlation between the measurement of fattening using ultrasound and measurements obtained using BIA (r = 0.81, *P* < 0.0001, Fig 2C and Table 7), by carcass analysis (r = 0.92, *P* < 0.0001, Fig 2D and Table 7), by X-ray computerised tomography scanner (r = 0.87, *P* < 0.0001, Fig 2E and Table 7) and by measuring the weight of abdominal fat (r = 0.86, *P* < 0.0001, Fig 2F and Table 7). This suggests that fat ultrasonographic examination represents a reliable method for evaluating fattening of hens. At week 39, the effect of the diet on animal growth was confirmed by measurement of the surface area of muscles (Pecto: 45.3 ± 5.04 cm^2^ *vs* 53.96 ± 3.23 cm^2^, *P* = 0.04; oyster muscle: 4.7 ± 0.26 cm^2^ *vs* 6.52 ± 0.23 cm^2^, *P* = 0.0004) (Fig 3A and 3B). However, no effect of fish oil supplementation was observed on muscle surface area.

**Table 7:** Pearson correlation coefficients (r) calculated between different methods used to assess fattening at 39 weeks. Values of r and significance of the correlations are indicated on the graphs. Correlations were considered as significant if *P* ≤ 0.05. BIA: bioelectrical impedance analysis, abd AT: abdominal adipose tissue.

		BIA	Carcass	Scanner	Weight of abd AT (n = 15 for each condition)
Ultrasound (n = 80 for each condition)	r	0.82	0.92	0.87	0.86
	*P*	< 0.0001	< 0.0001	< 0.0001	< 0.0001
BIA (n = 80 for each condition)	r		0.79	0.77	0.74
	*P*		< 0.0001	< 0.0001	< 0.0001
Carcass (n = 8 for each condition)	r			0.81	0.85
	*P*			0.001	< 0.0001
Scanner (n = 12 for each condition)	r				0.97
	*P*				< 0.0001

### Effect of restricted diet and fish oil supplementation on plasma metabolic factor concentrations from 3 to 39 weeks

**Fig 4:**
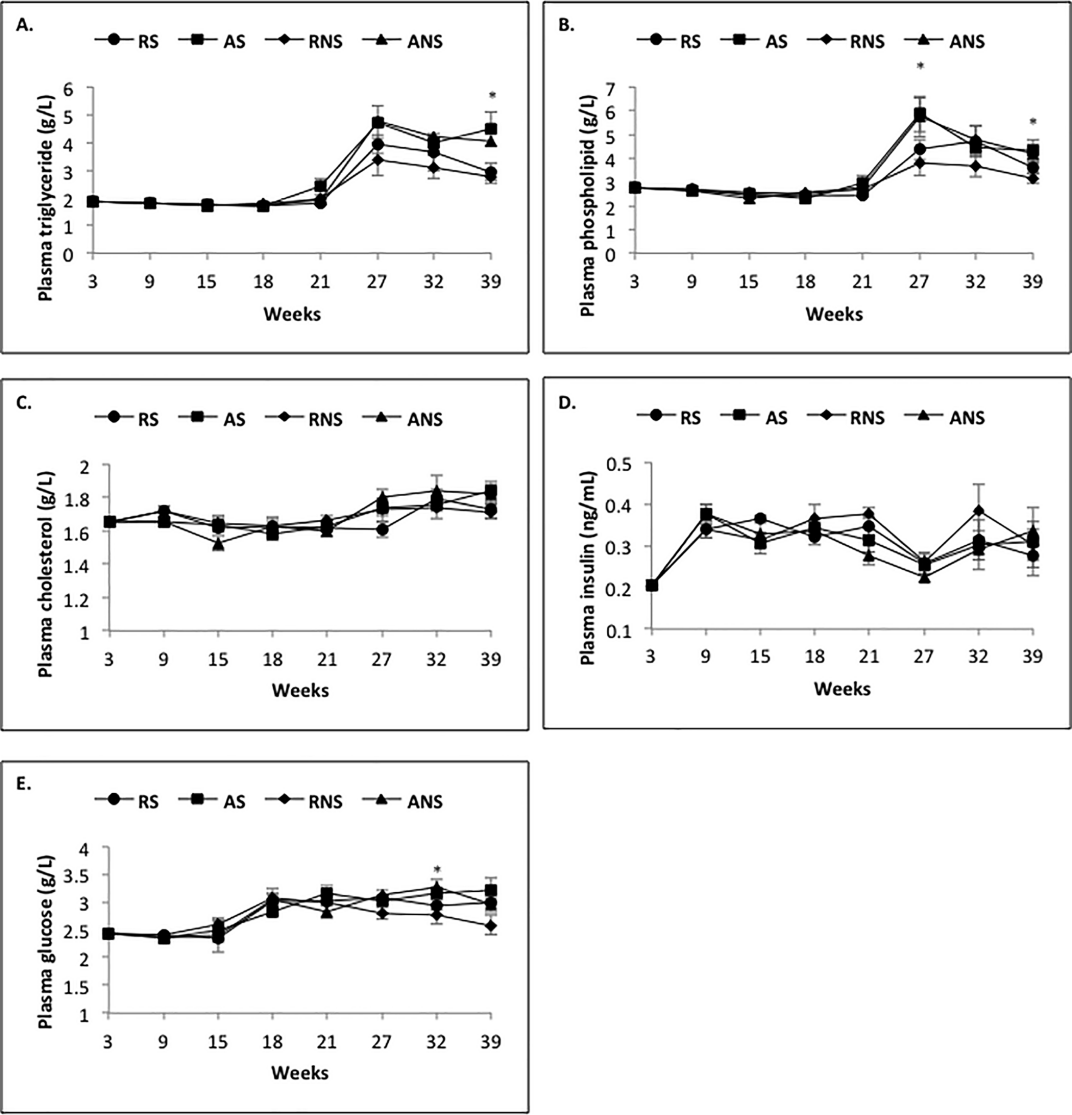
**Plasma concentrations of triglycerides (A), phospholipids (B), cholesterol (C), insulin (D) and glucose (E) in broiler hens fed *ad libitum* or with restricted diet, with or without fish oil supplementation.** Blood samples were collected every 6 weeks from week 3 to week 39, in the morning and before diet distribution. RS: animals fed with restricted and supplemented diet (n = 8), AS: animals fed *ad libitum* with supplemented diet (n = 8), RNS: animals fed with restricted and unsupplemented diet (n = 8), ANS: animals fed *ad libitum* with unsupplemented diet (n = 8). Results are presented as lsmeans ± s.e.m. *P < 0.05 (diet effect) and°*P* < 0.05 (fish oil supplementation effect).

As shown in Table 8 and Fig 4, all the plasma concentration profiles of biochemical parameters varied significantly with time (*P* < 0.0001), while plasma concentrations of triglyceride (*P* < 0.0001), phospholipid (*P* = 0.0003) and glucose (*P* = 0.02) were also decreased by the Rt diet and no effect of fish oil supplementation was observed. The Rt diet decreased the plasma triglyceride and glucose concentrations during both growing (triglyceride: 1.78 ± 0.01 g/l *vs* 1.88 ± 0.03 g/l, *P* = 0.001; glucose: 2.36 ± 0.03 g/l *vs* 2.46 ± 0.04 g/l, *P* = 0.04) and laying periods (triglyceride: 2.94 ± 0.23 g/l *vs* 3.80 ± 0.31 g/l, *P* = 0.002; glucose: 2.90 ± 0.05 g/l *vs* 3.09 ± 0.07 g/l, *P* = 0.003) (Tables 4 and 5) compared to Ad diet. The effects of the diet appeared more precisely at week 39 for plasma triglyceride concentrations (2.80 ± 0.04 g/l *vs* 4.25 ± 0.06 g/l, *P* = 0.003) and at week 32 for plasma glucose concentrations (2.85 ± 0.03 g/l *vs* 3.25 ± 0.04 g/l, *P* = 0.04) (Fig 4A and 4E). The effect of the Rt diet on plasma phospholipid concentrations was noted during the laying period (3.56 ± 0.23 g/l *vs* 4.37 ± 0.31 g/l, *P* = 0.003) (Table 8) and more specifically at week 27 (4.07 ± 0.50 g/l *vs* 5.79 ± 0.85 g/l, *P* = 0.01) and week 39 (3.40 ± 0.30 g/l *vs* 4.25 ± 0.45 g/l, *P* = 0.003) (Fig 4B). Hence, the various diets had different effects depending on the time-point and the biochemical parameters studied.

**Table 8:** Effect of week, diet and fish oil supplementation on plasma metabolic factors concentrations of hens fed *ad libitum* (Ad) or restricted (Rt) either with (Supp) or without (Crtl) fish oil supplementation (n = 8 for each condition).

Periods (weeks)			Triglyceride (g/L)	Phospholipid (g/L)	Cholesterol (g/L)	Glucose (g/L)	Insulin (ng/mL)
0 to 39	Ad	Ctrl	2.76 ± 0.21 ᵃ	3.44 ± 0.20 ᵃ	1.70 ± 0.02	2.80 ± 0.06 ᵃᵇ	0.29 ± 0.01
		Supp	2.86 ± 0.188 ᵇ	3.51 ± 0.19 ᵇ	1.69 ± 0.02	2.85 ± 0.06 ᵃ	0.30 ± 0.02
	Rt	Ctrl	2.28 ± 0.12 ᵃ	2.94 ± 0.11 ᵃ	1.68 ± 0.02	2.69 ± 0.05 ᵇ	0.33 ± 0.01
		Supp	2.42 ± 0.15 ᵃᵇ	3.17 ± 0.15 ᵃᵇ	1.67 ± 0.02	2.77 ± 0.05 ᵃᵇ	0.30 ± 0.01
	*P*	diet	**< 0.0001**	**0.0003**	0.21	**0.02**	0.077
		supp	0.32	0.25	0.63	0.23	0.60
		week	**< 0.0001**	**< 0.0001**	**< 0.0001**	**< 0.0001**	**< 0.0001**
		diet*supp	0.85	0.45	0.85	0.57	0.17
		diet*week	**0.01**	**0.002**	**0.03**	**0.04**	0.11
		supp*week	0.87	0.95	0.73	0.14	0.95
3 to 9	Ad	Ctrl	1.88 ± 0.03	2.78 ± 0.08	1.68 ± 0.03	2.46 ± 0.04	0.27 ± 0.02
Growing	Rt	Ctrl	1.78 ± 0.01	2.63 ± 0.05	1.67 ± 0.02	2.36 ± 0.03	0.29 ± 0.02
	*P*	diet	**0.001**	0.12	0.72	**0.04**	0.57
9 to 18	Ad	Ctrl	1.77 ± 0.02	2.49 ± 0.06	1.60 ± 0.03	2.61 ± 0.12	0.34 ± 0.01
Growing		Supp	1.76 ± 0.02	2.46 ± 0.05	1.63 ± 0.02	2.55 ± 0.07	0.35 ± 0.02
	Rt	Ctrl	1.77 ± 0.02	2.57 ± 0.05	1.67 ± 0.02	2.69 ± 0.07	0.35 ± 0.02
		Supp	1.74 ± 0.01	2.55 ± 0.08	1.66 ± 0.03	2.59 ± 0.10	0.33 ± 0.01
	*P*	diet	0.50	0.17	0.06	0.48	0.83
		supp	0.30	0.71	0.70	0.39	0.81
		diet*supp	0.60	0.92	0.57	0.83	0.53
18 to 21	Ad	Ctrl	1.86 ± 0.05	2.65 ± 0.07	1.61 ± 0.02	2.93 ± 0.08	0.30 ± 0.02 ᵃ
Before		Supp	2.05 ± 0.17	2.62 ± 0.16	1.60 ± 0.03	2.99 ± 0.09	0.33 ± 0.02 ᵃᵇ
laying	Rt	Ctrl	1.83 ± 0.04	2.58 ± 0.06	1.65 ± 0.02	3.04 ± 0.04	0.37 ± 0.02 ᵇ
		Supp	1.77 ± 0.02	2.44 ± 0.08	1.62 ± 0.03	3.03 ± 0.12	0.34 ± 0.02 ᵃᵇ
	*P*	diet	0.09	0.21	0.30	0.40	0.03
		supp	0.51	0.40	0.47	0.78	0.62
		diet*supp	0.17	0.58	0.73	0.68	0.08
21 to 39	Ad	Ctrl	3.72 ± 0.34 ᵃᶜ	4.34 ± 0.33 ᵃ	1.77 ± 0.03	3.03 ± 0.07 ᵃ	0.28 ± 0.01
Laying		Supp	3.89 ± 0.27 ᵃ	4.40 ± 0.29 ᵃ	1.74 ± 0.03	3.14 ± 0.07 ᵃ	0.30 ± 0.03
	Rt	Ctrl	2.79 ± 0.21 ᵇ	3.33 ± 0.20 ᵇ	1.71 ± 0.03	2.79 ± 0.07 ᵇ	0.33 ± 0.02
		Supp	3.08 ± 0.24 ᵇᶜ	3.78 ± 0.25 ᵃᵇ	1.69 ± 0.03	3.01 ± 0.03 ᵃ	0.30 ± 0.01
	*P*	diet	**0.002**	**0.003**	0.06	**0.003**	0.18
		supp	0.39	0.36	0.39	0.008	0.59
		diet*supp	0.83	0.47	0.99	0.37	0.21

Results are presented as lsmeans ± SEM. P values of the effects of diet, supplementation, week and the interaction between diet and supplementation, diet and week, and supplementation and week were considered as significant if *P* ≤ 0.05. Different individual (a,b, c) or group (ab, bc) of letters with no letter in common in superscript indicate significant differences.

### Effect of restricted diet and fish oil supplementation on plasma adipokine concentrations from 3 to 39 weeks

**Fig 5:**
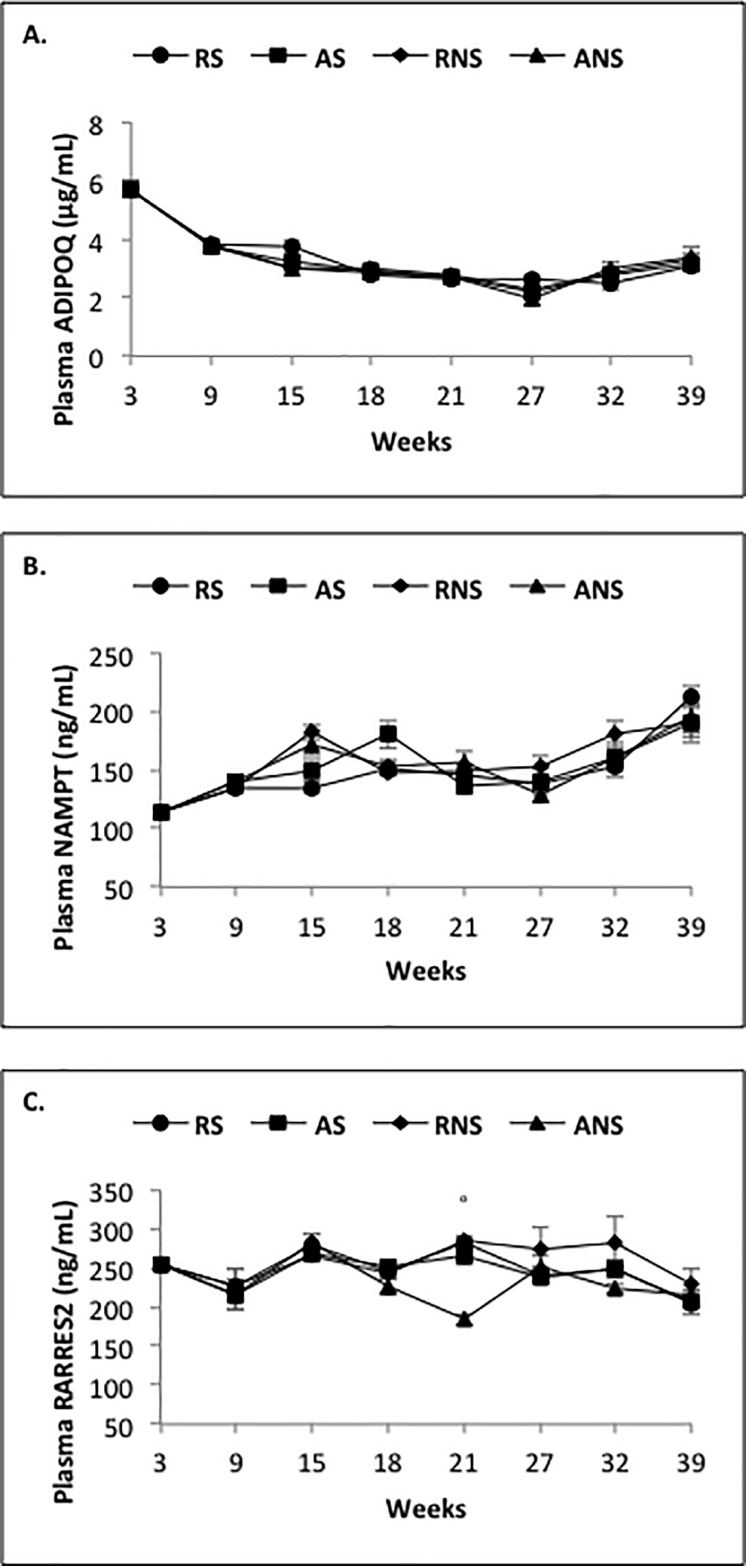
**Plasma concentrations of ADIPOQ (A), NAMPT (B) and RARRES2 in broiler hens fed *ad libitum* or with a restricted diet, with or without fish oil supplementation.** Blood samples were collected every 6 weeks from week 3 to week 39, in the morning and before diet distribution. RS: animals fed with restricted and supplemented diet (n = 8), AS: animals fed *ad libitum* with supplemented diet (n = 8), RNS: animals fed with restricted and unsupplemented diet (n = 8), ANS: animals fed *ad libitum* with unsupplemented diet (n = 8). Results are presented as lsmeans ± s.e.m. *P < 0.05 (diet effect) and°*P* < 0.05 (fish oil supplementation effect).

As shown for plasma concentrations of biochemical parameters, we observed a significant but weak effect (*P* < 0.0001) on plasma ADIPOQ, NAMPT and RARRES2 concentrations (Table 9). Plasma ADIPOQ concentrations decreased from week 3 to 27 and increased slightly from week 27 to 39 (Fig 5A). Plasma NAMPT concentrations increased from week 3 to 18, then decreased from week 18 to 27 and increased from week 27 to 39 (Fig 5B). Plasma RARRES2 concentrations alternately decreased and increased from week 3 to 18, then remained stable from week 18 to 32 and decreased from week 32 to 39 (Fig 5C). Unlike plasma NAMPT and RARRES2 concentrations, we showed that plasma ADIPOQ concentrations were decreased by the Rt diet (3.3 ± 0.14 μg/ml *vs* 3.43 ± 0.19 μg/ml, *P* = 0.003) and especially during the growing period (week 3 to 9: 4.25 ± 0.13 μg/ml *vs* 5.26 ± 0.3 μg/ml, *P* = 0.004) (Table 9). Fish oil supplementation did not alter plasma ADIPOQ and NAMPT concentrations, whereas it decreased those of RARRES2 (252.92 ± 6.61 ng/ml *vs* 238.19 ± 5.88 ng/ml, *P* = 0.003) (Table 9). Regarding to the different time periods, plasma concentrations of RARRES2 decreased during the growing period (week 9 to 18: 252.92 ± 6.61 ng/ml *vs* 238.19 ± 5.88 ng/ml, *P* = 0.0003), whereas they increased before the laying period (252.92 ± 6.61 ng/ml *vs* 238.19 ± 5.88 ng/ml, *P* < 0.0001) in supplemented animals (Table 9).

**Table 9:** Effect of week, diet and fish oil supplementation on plasma adipokine concentrations of hens fed *ad libitum* (Ad) or restricted (Rt) either with (Supp) or without (Crtl) fish oil supplementation (n = 8 for each conditions).

Periods (weeks)			ADIPOQ (μg/mL)	NAMPT (ng/mL)	RARRES2 (ng/mL)
0 to 39	Ad	Ctrl	3.44 ± 0.20 ᵃ	150.62 ± 4.19	243.45 ± 5.61 ᵃ
		Supp	3.41 ± 0.17 ᵃ	150.46 ± 4.74	238.36 ± 6.15 ᵃ
	Rt	Ctrl	3.21 ± 0.10 ᵃ	157.47 ± 3.95	262.39 ± 7.61 ᵇ
		Supp	3.24 ± 0.17 ᵃ	147.98 ± 4.09	238.02 ± 5.60 ᵃ
	*P*	diet	**0.003**	0.55	0.06
		supp	0.99	0.09	**0.003**
		week	**< 0.0001**	**< 0.0001**	**< 0.0001**
		diet*supp	0.66	0.16	**0.05**
		diet*week	**< 0.0001**	0.18	**< 0.0001**
		supp*week	0.09	**0.005**	**< 0.0001**
3 to 9	Ad	Ctrl	5.26 ± 0.3	123.43 ± 3.85	247.84 ± 11.73
Growing	Rt	Ctrl	4.25 ± 0.13	126.87 ± 2.94	229.09 ± 11.95
	*P*	diet	**0.004**	0.49	0.22
9 to 18	Ad	Ctrl	3.16 ± 0.10	155.15 ± 4.19	264.30 ± 8.42 ᵃ
Growing		Supp	3.32 ± 0.10	156.60 ± 6.44	223.03 ± 11.79 ᵇ
	Rt	Ctrl	3.33 ± 0.10	155.45 ± 4.98	266.98 ± 9.59 ᵃ
		Supp	3.42 ± 0.13	138.91 ± 3.34	230.13 ± 11.34 ᵇ
	*P*	diet	0.24	0.08	0.64
		supp	0.25	0.12	**0.0003**
		diet*supp	0.77	0.07	0.83
18 to 21	Ad	Ctrl	2.77 ± 0.06	154.63 ± 5.32	205.51 ± 8.51 ᵃ
Before		Supp	2.80 ± 0.07	158.37 ± 8.73	258.27 ± 6.48 ᵇ
laying	Rt	Ctrl	2.89 ± 0.07	148.22 ± 3.72	264.88 ± 7.25 ᵇ
		Supp	2.73 ± 0.07	147.99 ± 5.54	264.22 ± 5.99 ᵇ
	*P*	diet	0.68	0.17	< 0.0001
		supp	0.37	0.77	**0.0005**
		diet*supp	0.14	0.75	**0.004**
21 to 39	Ad	Ctrl	2.76 ± 0.15	158.63 ± 6.54	218.66 ± 5.95 ᵃ
Laying		Supp	2.70 ± 0.10	156.84 ± 7.05	239.47 ± 6.93 ᵃᶜ
	Rt	Ctrl	2.80 ± 0.12	167.90 ± 5.85	268.53 ± 11.89 ᵇ
		Supp	2.70 ± 0.09	162.28 ± 6.57	244.20 ± 6.49 ᶜ
	*P*	diet	0.89	0.26	0.001
		supp	0.49	0.57	0.83
		diet*supp	0.84	0.77	0.007

Results are presented as lsmeans ± SEM. P values of the effects of diet, supplementation, week and the interaction between diet and supplementation, diet and week, and supplementation and week were considered as significant if *P* ≤ 0.05. Different individual (a,b, c) or group (ac) of letters with no letter in common in superscript indicate significant differences.

More precisely, the effect observed before laying appeared at week 21 (235 ± 7 ng/ml *vs* 273 ± 8.5 ng/ml, P < 0.0001) (Fig 5C). As shown in Table 10, we observed that the plasma levels of all the adipokines tested were negatively correlated with those of triglyceride (ADIPOQ: r = - 0.41, *P* < 0.0001; NAMPT: r = - 0.19, *P* = 0.01; RARRES2: r = - 0.35, *P* < 0.0001) and phospholipid (ADIPOQ: r = - 0.45, *P* < 0.0001; NAMPT: r = - 0.25, *P* = 0.001; RARRES2: r = - 0.31, *P* < 0.0001) and positively correlated with plasma insulin concentrations (ADIPOQ: r = 0.38, *P* < 0.0001; NAMPT: r = 0.39, *P* < 0.0001; RARRES2: r = 0.51, *P* < 0.0001) (Table 10). We also showed a significant negative correlation between plasma ADIPOQ and RARRES2 concentrations and plasma cholesterol (ADIPOQ: r = - 0.22, *P* = 0.003; RARRES2: r = - 0.34, *P* < 0.0001) or glucose concentrations (ADIPOQ: r = - 0.28, *P* = 0.0001; RARRES2: r = - 0.34, *P* < 0.0001). In addition, we observed a positive correlation between plasma ADIPOQ concentrations with those of RARRES2 (r = 0.20, *P* = 0.01) and NAMPT (r = 0.43, *P* < 0.0001). We also found a negative correlation between plasma levels of RARRES2 and the percentage of fat mass evaluated by BIA (r = - 0.38, *P* < 0.0001) and ultrasound (r = - 0.28, *P* = 0.0002; Table 10). Hence, adipokines may reflect the metabolic status of animals depending on the time period.

**Table 10:** Pearson correlation coefficients (r) calculated between plasma metabolites and hormones (triglyceride, phospholipid, cholesterol, insulin, glucose, ADIPOQ, RARRES2 and NAMPT) and % of fat (BIA, ultrasound) during the period from 3 to 39 weeks.

		ADIPOQ	RARRES2	NAMPT
Triglyceride	r	- 0.41	- 0.35	- 0.19
	*P*	< 0.0001	< 0.0001	0.01
Phospholipid	r	- 0.45	- 0.31	- 0.25
	*P*	< 0.0001	< 0.0001	0.001
Cholesterol	r	- 0.22	- 0.34	- 0.08
	*P*	0.003	< 0.0001	0.31
Insulin	r	0.38	0.51	0.39
	*P*	< 0.0001	< 0.0001	< 0.0001
Glucose	r	- 0.28	- 0.34	- 0.11
	*P*	0.0001	< 0.0001	0.16
% fat (BIA)	r	n- 0.08	n- 0.38	0.14
	*P*	0.26	< 0.0001	0.06
% fat (ultrasound)	r	0.004	- 0.28	0.02
	*P*	0.95	0.0002	0.76
NAMPT	r	0.43	- 0.01	
	*P*	< 0.0001	0.93	
RARRES2	r	0.20		
	*P*	0.01		

Values of r and significance of the correlations are indicated on the graphs. Correlations were considered as significant if *P* ≤ 0.05.

### Effect of restricted diet and fish oil supplementation on expression of lipid metabolism factors in liver, muscle and adipose tissues at 39 weeks

We chose the genes involved in different processes of lipid and glucose metabolism, including lipogenesis (*FASN*: fatty acid synthase, *PPARG*: peroxisome proliferator-activated receptor gamma), fatty acid transport (*CD36*: cluster of differentiation 36, *FATP1*: fatty acid transport protein 1), glucose transport (*GLUT8*: glucose carrier 8) and fatty acid transport (*FFAR2*: free fatty acid receptor 2, *FFAR4*: free fatty acid receptor 4).

No effect of the Rt diet was observed in the liver, irrespective of the gene studied, whereas fish oil supplementation increased the expression of *FASN* (0.22 ± 0.04 *vs* 0.62 ± 0.15, *P* = 0.02), *FFAR4* (0.004 ± 0.0007 *vs* 0.008 ± 0.001, *P* = 0.01) and *GLUT8* (0.03 ± 0.002 *vs* 0.04 ± 0.005, *P* = 0.05) and decreased those of *FATP1* (0.024 ± 0.002 *vs* 0.020 ± 0.002, *P* = 0.04) (Table 11). In Pecto, the Rt diet increased the expression of *PPARG* (0.009 ± 0.0007 *vs* 0.008 ± 0.001, *P = 0*.*05*) and *FFAR4 (*0.002 ± 0.0001 *vs* 0.001 ± 0.0001, *P = 0*.*01)* and decreased the expression of *CD36* (0.77 ± 0.09 *vs* 1.06 ± 0.12, *P* = 0.05). On the other hand, fish oil supplementation decreased the expression of *FATP1* (2.05 ± 0.37 *vs* 0.85 ± 0.10, *P* = 0.01) and *CD36* (1.08 ± 0.13 *vs* 0.76 ± 0.07, *P* = 0.04) and increased those of *FFAR4* (0.001 ± 0.0001 *vs* 0.002 ± 0.0001, *P* = 0.001) (Table 11). In AT abd, the Rt diet only decreased the expression of *FASN* (0.02 ± 0.003 *vs* 0.05 ± 0.02, *P* = 0.05), whereas fish oil supplementation decreased the expression of *FASN* (0.05 ± 0.02 *vs* 0.01 ± 0.004, *P* = 0.04) and increased those of *CD36* (0.19 ± 0.06 *vs* 0.31 ± 0.006, *P* = 0.05) (Table 11). In AT sc, the Rt diet increased the expression of *FATP1* (0.61 ± 0.13 *vs* 0.12 ± 0.04, *P* = 0.001), *PPARG* (1.59 ± 0.24 *vs* 0.87 ± 0.22, *P* = 0.03) and *GLUT8* (0.56 ± 0.11 *vs* 0.20 ± 0.03, *P* = 0.01), and no effect of fish oil supplementation was detected (Table 11).

**Table 11:** mRNA expression of metabolic factors (*FASN*, *FATP1*, *PPARG*, *FFAR4*, *GLUT8* and *CD36*) in liver, pectoralis major (Pectco), abdominal (abd) and subcutaneous (sc) adipose tissue (AT).

	Diet	Ad		Rt				
Tissue	Supp	Ctrl (ANS)	Supp (AS)	Ctrl (RNS)	Supp (RS)	Diet	Supp	Diet*Supp
Liver	FASN	0.225 ± 0.053 ᵃ	0.575 ± 0.192 ᵃᵇ	0.225 ± 0.074 ᵃᵇ	0.671 ± 0.249 ᵇ	0.76	**0.02**	0.77
	FATP1	0.025 ± 0.003 ᵃ	0.018 ± 0.002 ᵇ	0.023 ± 0.001 ᵃᵇ	0.021 ± 0.003 ᵃᵇ	0.78	**0.04**	0.25
	PPARg	0.003 ± 0.001	0.006 ± 0.002	0.003 ± 0.001	0.004 ± 0.0003	0.42	0.14	0.57
	FFAR4	0.006 ± 0.001 ᵃᶜ	0.006 ± 0.001 ᵇᶜ	0.002 ± 0.0005 ᵃ	0.010 ± 0.002 ᵇ	0.94	**0.01**	**0.01**
	Glut8	0.029 ± 0.003 ᵃᵇ	0.033 ± 0.003 ᵃᵇ	0.026 ± 0.003 ᵃ	0.044 ± 0.008 ᵇ	0.45	**0.05**	0.26
	CD36	0.359 ± 0.058	0.355 ± 0.067	0.373 ± 0.031	0.305 ± 0.044	0.73	0.50	0.55
Pecto	FASN	0.255 ± 0.035	0.207 ± 0.025	0.194 ± 0.013	0.295 ± 0.057	0.71	0.48	**0.06**
	FATP1	2.562 ± 0.567 ᵃ	0.793 ± 0.141 ᵇ	1.451 ± 0.360 ᵃ	0.931 ± 0.142 ᵃ	0.21	**0.01**	0.12
	PPARg	0.009 ± 0.001	0.007 ± 0.001	0.008 ± 0.0004	0.011 ± 0.001	**0.05**	0.85	**0.005**
	FFAR4	0.002 ± 0.0002 ᵃ	0.001 ± 0.00005 ᵇ	0.001 ± 0.0001 ᵇ	0.003 ± 0.0002 ᶜ	**0.01**	**0.001**	**<0.0001**
	Glut8	0.086 ± 0.017	0.152 ± 0.031	0.153 ± 0.039	0.032 ± 0.003	0.35	0.34	**0.003**
	CD36	1.250 ± 0.186 ᵃ	0.861 ± 0.098 ᵃᵇ	0.884 ± 0.153 ᵃᵇ	0.650 ± 0.085 ᵇ	**0.05**	**0.04**	0.58
AT abd	FASN	0.090 ± 0.043 ᵃ	0.015 ± 0.009 ᵇ	0.017 ± 0.006 ᵇ	0.008 ± 0.002 ᵇ	**0.05**	**0.04**	0.10
	FATP1	0.014 ± 0.008	0.004 ± 0.001	0.005 ± 0.0003	0.006 ± 0.002	0.34	0.27	0.17
	PPARg	0.525 ± 0.294	0.087 ± 0.035	0.059 ± 0.023	0.046 ± 0.012	0.08	0.11	0.14
	FFAR4	0.005 ± 0.003 ᵃ	0.0002 ± 0.00003 ᵇ	0.0002 ± 0.00004 ᵇ	0.0004 ± 0.0001 ᵇ	0.07	0.07	**0.05**
	Glut8	0.006 ± 0.002	0.004 ± 0.0004	0.004 ± 0.001	0.002 ± 0.0003	0.07	0.11	0.89
	CD36	0.171 ± 0.064	0.347 ± 0.063	0.214 ± 0.050	0.275 ± 0.057	0.80	**0.05**	0.34
AT sc	FASN	0.502 ± 0.073	1.227 ± 0.567	1.656 ± 0.278	1.236 ± 0.399	0.15	0.70	0.15
	FATP1	0.183 ± 0.052 ᵃᶜ	0.049 ± 0.029 ᵃ	0.724 ± 0.202 ᵇ	0.495 ± 0.149 ᵇᶜ	**0.001**	0.19	0.72
	PPARg	0.818 ± 0.212 ᵃ	0.912 ± 0.406 ᵃᵇ	1.800 ± 0.182 ᵇ	1.414 ± 0.419 ᵃᵇ	**0.03**	0.66	0.48
	FFAR4	0.002 ± 0.001 ᵃ	0.002 ± 0.0004 ᵃ	0.002 ± 0.0002 ᵃ	0.006 ± 0.002 ᵇ	0.10	0.10	0.06
	Glut8	0.231 ± 0.038 ᵃᶜ	0.158 ± 0.036 ᵃ	0.523 ± 0.070 ᵇᶜ	0.604 ± 0.239 ᵇ	**0.01**	0.98	0.54
	CD36	0.004 ± 0.001	0.009 ± 0.006	0.007 ± 0.003	0.003 ± 0.001	0.63	0.95	0.20

Results are presented as lsmeans ± SEM. P values of the effects of diet, supplementation and the interaction between diet and supplementation were considered as significant if *P* ≤ 0.05. Different letters in superscript (a,b and c) or group (ab, bc) of letters with no letter in common in superscript indicate significant differences. Ad: *ad libitum*, Rt: restricted, Supp: supplemented, Ctrl: control unsupplemented, ANS: *ad libitum* unsupplemented (n = 8), AS: *ad libitum* supplemented (n = 8), RNS: restricted unsupplemented (n = 8), RS: restricted supplemented (n = 8).

### Effect of restricted diet and fish oil supplementation on expression of adipokines and their receptors in liver, muscle and adipose tissues at 39 weeks

In liver, the Rt diet decreased the expression of *RARRES2* (2.02 ± 0.17 *vs* 2.65 ± 0.25, *P* = 0.04) and *NAMPT* (0.18 ± 0.01 *vs* 0.30 ± 0.04, *P* = 0.03). Fish oil supplementation decreased the expression of *ADIPOR1* (0.18 ± 0.02 *vs* 0.13 ± 0.01, *P* = 0.03) (Table 12). In Pecto, the Rt diet had no effect on any of the studied genes, whereas fish oil supplementation decreased the expression of *ADIPOQ* (0.18 ± 0.002 *vs* 0.03 ± 0.006, *P* = 0.001) and *CCRL2* (0.01 ± 0.001 *vs* 0.007 ± 0.0007, *P* = 0.001) and increased those of *ADIPOR1* (0.24 ± 0.03 *vs* 0.31 ± 0.04, *P* = 0.05) (Table 12). In AT abd, the Rt diet reduced the expression of *RARRES2* (0.05 ± 0.004 *vs* 0.08 ± 0.01, *P* = 0.05) and *CMKLR1* (0.003 ± 0.0007 *vs* 0.006 ± 0.001, *P* = 0.01), while fish oil supplementation had no effect (Table 12). In AT sc, the Rt diet increased the expression of *ADIPOQ* (1.64 ± 0.26 *vs* 0.74 ± 0.13, *P* = 0.002), *CMKLR1* (0.35 ± 0.07 *vs* 0.08 ± 0.03, *P* = 0.0001) and *CCRL2* (1.04 ± 0.31 vs 0.21 ± 0.07, *P* = 0.002). In contrast, fish oil supplementation decreased the expression of *ADIPOQ* (1.58 ± 0.24 *vs* 0.75 ± 0.17, *P* = 0.004) and *CMKLR1* (0.29 ± 0.06 *vs* 0.14 ± 0.04, *P* = 0.01) (Table 12).

**Table 12:** mRNA expression of adipokines (*ADIPOQ*, *RARRES2* and *NAMPT*) and adipokine receptors (*ADIPOR1*, *ADIPOR2*, *CMKLR1*, *CCRL2*) in liver, pectoralis major (Pectco), abdominal (abd) and subcutaneous (sc) adipose tissue (AT).

		Diet	Ad		Rt			*P*	
Tissue		Supp	Ctrl (ANS)	Supp (AS)	Ctrl (RNS)	Supp (RS)	Diet	Supp	Diet*Supp
Liver	Adipokines	*ADIPOQ*	0.001 ± 0.0002	0.002 ± 0.001	0.001 ± 0.0001	0.002 ± 0.0004	0.53	0.06	0.66
		*RARRES2*	2.920 ± 0.367 ᵃ	2.414 ± 0.349 ᵃᵇ	1.870 ± 0.206 ᵇ	2.156 ± 0.277 ᵃᵇ	**0.04**	0.73	0.21
		*NAMPT*	0.351 ± 0.087 ᵃ	0.253 ± 0.033 ᵃᵇ	0.163 ± 0.012 ᵃ	0.218 ± 0.035 ᵃᵇ	**0.03**	0.66	0.12
	Adipokine receptors	*ADIPOR1*	0.174 ± 0.019 ᵃᵇ	0.118 ± 0.011 ᵃ	0.176 ± 0.029 ᵇ	0.139 ± 0.015 ᵃᵇ	0.56	**0.03**	0.65
		*ADIPOR2*	0.311 ± 0.048	0.288 ± 0.045	0.363 ± 0.051	0.268 ± 0.039	0.73	0.21	0.43
		*CMKLR1*	0.003 ± 0.001	0.005 ± 0.001	0.002 ± 0.00016	0.003 ± 0.001	0.17	0.15	1.00
		*CCRL2*	0.016 ± 0.002	0.013 ± 0.002	0.016 ± 0.002	0.014 ± 0.003	1.00	0.34	0.79
Pecto	Adipokines	*ADIPOQ*	0.015 ± 0.002 ᵃ	0.029 ± 0.006 ᵇ	0.017 ± 0.003 ᵃ	0.035 ± 0.006 ᵇ	0.32	**0.001**	0.62
		*RARRES2*	0.036 ± 0.002	0.033 ± 0.004	0.042 ± 0.006	0.030 ± 0.003	0.76	0.07	0.22
		*NAMPT*	0.143 ± 0.018	0.190 ± 0.032	0.159 ± 0.008	0.107 ± 0.027	0.17	0.91	**0.05**
	Adipokine receptors	*ADIPOR1*	0.268 ± 0.023 ᵃ	0.323 ± 0.035 ᵇ	0.206 ± 0.033 ᵃ	0.294 ± 0.044 ᵇ	0.19	**0.05**	0.64
		*ADIPOR2*	0.901 ± 0.130	1 ± 0.139	0.554 ± 0.107	0.909 ± 0.121	0.11	0.09	0.33
		*CMKLR1*	0.004 ± 0.001	0.004 ± 0.001	0.005 ± 0.0004	0.008 ± 0.002	0.08	0.12	0.11
		*CCRL2*	0.015 ± 0.002	0.006 ± 0.0004	0.010 ± 0.001	0.008 ± 0.001	0.45	**0.001**	**0.03**
AT abd	Adipokines	*ADIPOQ*	0.015 ± 0.008	0.065 ± 0.031	0.025 ± 0.015	0.018 ± 0.003	0.33	0.25	0.14
		*RARRES2*	0.092 ± 0.021 ᵃ	0.061 ± 0.006 ᵃᵇ	0.056 ± 0.007 ᵇ	0.051 ± 0.004 ᵇ	**0.05**	0.12	0.24
		*NAMPT*	335.246 ± 166.026	80.735 ± 44.187	83.475 ± 43.725	47.629 ± 26.444	0.12	0.11	0.22
	Adipokine receptors	*ADIPOR1*	0.006 ± 0.002	0.03 ± 0.005	0.022 ± 0.006	0.011 ± 0.003	0.78	0.19	**0.002**
		*ADIPOR2*	0.004 ± 0.002	0.025 ± 0.004	0.024 ± 0.008	0.004 ± 0.002	0.89	0.96	**0.01**
		*CMKLR1*	0.005 ± 0.001 ᵃᵇ	0.006 ± 0.001 ᵇ	0.002 ± 0.0004 ᵃ	0.003 ± 0.001 ᵃ	**0.01**	0.47	0.76
		*CCRL2*	0.064 ± 0.027	0.013 ± 0.004	0.012 ± 0.001	0.008 ± 0.002	0.08	0.09	0.14
AT sc	Adipokines	*ADIPOQ*	1.058 ± 0.161 ᵃ	0.379 ± 0.113 ᵃ	2.092 ± 0.387 ᵇ	1.126 ± 0.244 ᵃ	**0.002**	**0.004**	0.58
		*RARRES2*	0.223 ± 0.031	0.289 ± 0.092	0.459 ± 0.067	0.460 ± 0.163	0.06	0.75	0.76
		*NAMPT*	14.604 ± 5.458	15.039 ± 7.085	32.553 ± 9.681	14.932 ± 3.992	0.21	0.23	0.21
	Adipokine receptors	*ADIPOR1*	0.021 ± 0.005	0.024 ± 0.005	0.023 ± 0.004	0.023 ± 0.006	0.90	0.79	0.80
		*ADIPOR2*	0.008 ± 0.003	0.037 ± 0.011	0.03 ± 0.011	0.032 ± 0.010	0.41	0.13	0.16
		*CMKLR1*	0.119 ± 0.027 ᵃᵇ	0.044 ± 0.026 ᵇ	0.465 ± 0.091 ᶜ	0.228 ± 0.057 ᵃ	**0.0001**	**0.01**	0.18
		*CCRL2*	0.270 ± 0.046 ᵃᶜ	0.150 ± 0.103 ᵃ	1.150 ± 0.286 ᵇ	0.937 ± 0.324 ᵇᶜ	**0.002**	0.49	0.85

Results are presented as lsmeans ± SEM. P values of the effects of diet, supplementation and the interaction between diet and supplementation were considered as significant if *P* ≤ 0.05. Different letters in superscript (a,b and c) or group (ab, bc) of letters with no letter in common in superscript indicate significant differences.Ad: *ad libitum*, Rt: restricted, Supp: supplemented, Ctrl: control unsupplemented, ANS: *ad libitum* unsupplemented (n = 8), AS: *ad libitum* supplemented (n = 8), RNS: restricted unsupplemented (n = 8), RS: restricted supplemented (n = 8).

### Correlation between expression of adipokines and different metabolic parameters in metabolic tissues at 39 weeks

As shown in Table 13, the expression of *ADIPOQ* in liver tissue was correlated with the expression of *PPARG* (r = 0.49, *P* = 0.02), *GLUT8* (r = 0.40, *P* = 0.05) and *CD36* (r = - 0.68, *P* < 0.0001), while the expression of *RARRES2* was correlated with those of *PPARG* (r = - 0.49, *P* = 0.01) and *CD36* (r = 0.59, *P* = 0.001). We also noted that the expression of *NAMPT* was only correlated with *CD36* (r = 0.40, *P* = 0.04) (Table 13). In Pecto, the expression of *NAMPT* tended to be correlated with the expression of *PPARG* (r = - 0.40, *P* = 0.06) (Table 13). In AT abd, the expression of *NAMPT* was significantly correlated with the expression of all metabolic factors tested and the expression of *RARRES2* was correlated with those of *FASN* (r = 0.78, *P* < 0.0001), *PPARG* (r = 0.73, *P* < 0.0001) and *GLUT8* (r = 0.76, *P* < 0.0001) (Table 13). In AT sc, we observed that the expression of *RARRES2* and *ADIPOQ* were correlated with the expression of *FASN* (r = 0.82, *P* < 0.0001 and r = 0.62, *P* = 0.0002, respectively), *FATP1* (r = 0.77, *P* < 0.0001and r = 0.84, *P* < 0.0001, respectively), *PPARG* (r = 0.86, *P* < 0.0001 and r = 0.68, *P* < 0.0001, respectively) and *GLUT8* (r = 0.77, *P* < 0.0001 and r = 0.43, *P* = 0.02, respectively). Furthermore, the expression of *NAMPT* was correlated with the expression of *FASN* (r = 0.57, *P* = 0.001) and *PPARG* (r = 0.52, *P* = 0.004) (Table 13).

**Table 13:** Pearson correlation coefficients (r) calculated between metabolic factors (*FASN*, *FATP1*, *PPARG*, *FFAR4*, *FFAR2*, *GLUT8* and *CD36*) mRNA expression and adipokines (*RARRES2*, *NAMPT* and *ADIPOQ*) mRNA expression in liver, pectoralis major (Pecto), abdominal (abd) and subcutaneous (sc) adipose tissue (AT) at 39 weeks.

**Liver**														
	*FASN*		*FATP1*		*PPARG*		*FFAR4*		*FFAR2*		*GLUT8*		*CD36*	
	r	*P*	r	*P*	r	*P*	r	*P*	r	*P*	r	*P*	r	*P*
*RARRES2*	0.23	0.23	- 0.03	0.87	**- 0.49**	**0.01**	0.11	0.60	0.08	0.71	- 0.19	0.35	**0.59**	**0.001**
*NAMPT*	- 0.01	0.95	0.14	0.50	- 0.15	0.47	0.04	0.86	- 0.13	0.53	- 0.10	0.64	**0.40**	**0.04**
*ADIPOQ*	- 0.004	0.98	- 0.21	0.34	**0.49**	**0.02**	0.10	0.64	0.23	0.25	**0.40**	**0.05**	**- 0.68**	**< 0.0001**
**Pecto**														
	*FASN*		*FATP1*		*PPARG*		*FFAR4*		*FFAR2*		*GLUT8*		*CD36*	
	r	*P*	r	*P*	r	*P*	r	*P*	r	*P*	r	*P*	r	*P*
*RARRES2*	- 1.33	0.54	0.08	0.71	0.01	0.97	- 0.31	0.16	- 0.14	0.53	0.02	0.93	- 0.07	0.75
*NAMPT*	- 0.37	0.09	0.002	0.99	**- 0.40**	**0.06**	- 0.37	0.09	- 0.30	0.17	0.31	0.17	0.09	0.69
*ADIPOQ*	- 0.02	0.92	- 0.36	0.10	0.07	0.76	0.15	0.53	- 0.04	0.86	0.01	0.97	- 0.25	0.25
**AT abd**														
	*FASN*		*FATP1*		*PPARG*		*FFAR4*		*FFAR2*		*GLUT8*		*CD36*	
	r	*P*	r	*P*	r	*P*	r	*P*	r	*P*	r	*P*	r	*P*
*RARRES2*	**0.78**	**< 0.0001**	0.69	0.12	**0.73**	**< 0.0001**	0.07	0.79	0.47	0.03	**0.76**	**< 0.0001**	- 0.04	0.84
*NAMPT*	**0.78**	**< 0.0001**	**0.69**	**< 0.0001**	**0.46**	**0.02**	**0.57**	**0.01**	**0.44**	**0.04**	**0.73**	**< 0.0001**	**- 0.39**	**0.05**
*ADIPOQ*	0.17	0.42	0.36	0.08	0.09	0.68	0.02	0.95	0.18	0.44	0.01	0.96	- 0.23	0.27
**AT sc**														
	*FASN*		*FATP1*		*PPARG*		*FFAR4*		*FFAR2*		*GLUT8*		*CD36*	
	r	*P*	r	*P*	r	*P*	r	*P*	r	*P*	r	*P*	r	*P*
*RARRES2*	**0.82**	**< 0.0001**	**0.77**	**< 0.0001**	**0.86**	**< 0.0001**	0.15	0.47	0.13	0.55	**0.77**	**< 0.0001**	0.39	0.09
*NAMPT*	**0.57**	**0.001**	0.23	0.22	**0.52**	**0.004**	- 0.002	0.99	0.21	0.33	0.34	0.08	- 0.14	0.58
*ADIPOQ*	**0.62**	**0.0002**	**0.84**	**< 0.0001**	**0.68**	**< 0.0001**	- 0.03	0.89	0.21	0.32	**0.43**	**0.02**	0.05	0.82

Values of r and significance of the correlations are indicated on the graphs. Correlations were considered as significant if *P* ≤ 0.05.

### Correlations between adipokine levels in plasma and adipose tissue at 39 weeks

We did not find any correlation between adipokine expression in plasma and in ATs. However, we found a positive correlation between plasma ADIPOQ concentrations and the mRNA expression of *RARRES2* (r = 0.37, *P* = 0.05) and *NAMPT* (r = 0.38, *P* = 0.04) in AT abd, and between plasma NAMPT concentrations and the mRNA expression of *ADIPOQ* (r = - 0.40, *P* = 0.04) in AT abd (Table 14).

**Table 14:** Pearson correlation coefficients (r) calculated between plasma adipokine (RARRES2, NAMPT, ADIPOQ) concentrations and their mRNA expression in abdominal (abd) and subcutaneous (sc) adipose tissue (AT) at 39 weeks.

			Plasma		
			RARRES2	NAMPT	ADIPOQ
AT abd	RARRES2	r	- 0.20	0.06	**0.37**
		*P*	0.92	0.77	**0.05**
	NAMPT	r	0.06	0.14	**0.38**
		*P*	0.74	0.48	**0.04**
	ADIPOQ	r	- 0.10	**- 0.40**	- 0.14
		*P*	0.64	**0.04**	0.48
AT sc	RARRES2	r	- 0.22	0.01	0.19
		*P*	0.24	0.94	0.32
	NAMPT	r	- 0.08	- 0.14	- 0.03
		*P*	0.69	0.48	0.88
	ADIPOQ	r	- 0.09	- 0.17	0.11
		*P*	0.64	0.38	0.58

Values of r and significance of the correlations are indicated on the graphs. Correlations were considered as significant if *P* ≤ 0.05.

## Discussion

The present study shows that the quantity of food and its lipid composition influences body condition during development through the regulation of metabolic factors and adipokine expression in broiler breeder hens.

As expected, animals fed with the Rt diet were thinner, had less fat and smaller muscles than Ad animals. The state of the fattening was evaluated regularly throughout the study using multiple methods, including fat ultrasonographic examinations for the first time and by BIA, a non-invasive method. These results were positively correlated with those obtained by traditional methods, such as carcass analysis and weighing of abdominal adipose tissue. However, the calculations of BIA that were used do not yield results for animals less than 12 weeks old and we have no indication of the effect of the electrode clamps on the stress level in the animals. Thus, the use of ultrasound could provide a good technique for breeders to evaluate the fattening of their animals. Furthermore, the arrival on the market of new portable devices with very good image quality and for a reasonable price should favour the emergence of this technique.

As compared to mammals, the main site of lipogenesis in chicken is the liver [35] and the late reduction in fat mass in Rt animals was associated with different gene expression depending on the type of metabolic tissue. On the one hand, Rt diet-induced lipogenesis (*FASN*) was positively correlated with a decrease in *RARRES2* and its receptors *CMKLR1* in AT abd, and promoted the transport of fat (*FATP1*) and glucose (*GLUT8*) in AT sc associated with an increase in *ADIPOQ*, *CMKLR1* and *CCRL2* expression. However, in contrast to our findings, Tahmoorespur et al. (2010) found an increasing effect of food restriction on *ADIPOQ* mRNA expression in AT [36]. These may be explained by a transitory effect which depends on the duration of the food restriction. In the present study, we also observed that plasma RARRES2 was negatively correlated with the percentage of fat determined by BIA or ultrasound. These data are in contrast to those reported in humans. Indeed, plasma RARRES2 levels are increased in obese patients and reduce after bariatric surgery [37]. All of these results indicate that adipokines, especially RARRES2 and ADIPOQ, may be potential markers of fat mass in broiler breeder hens.

In the current study, we observed no fish oil-induced effects on growth, performance or fattening, whereas fish oil supplementation significantly increased the levels of omega 3 PUFAs in the adipose tissue and muscles. Fish oil is one of the major source of EPA, DPA and DHA. Previous studies have reported variable effects of fish oil on performance parameters [38] [39] [40] [41]. The lack of an effect of fish oil in the current study may be attributed to differences in the source of the fish oil, its composition, the proportion of supplement added to the diet and the diet composition. Conversely to plants, animals were not able to produce EPA and DHA despite the multiple studies that show their beneficial effects on inflammation [42] and insulin resistance [43] [44]. However, animals can metabolise EPA and DHA by a series of desaturation and elongation reactions of ALA, which are particularly active in the liver and to a lesser extent in other tissues [45]. Here, the low proportion of fish oil in the diet (1%) was sufficient to decrease the n-6/n-3 ratio (except in adipose tissue and thoracic limb), as previously demonstrated by Koppenol (2014) [11]. Supplementation with fish oil also led to a significant increase of EPA in egg yolks and in liver tissue, but had no effect on DHA levels in the liver. This may be explained by a reduction in the retroconversion of EPA in DHA *in vivo*, or by an effect of the nutritional status on desaturase enzyme leading to a complex hormonal retrocontrol. Similar results have been described by López-Ferrer et al. investigating the effect of supplying linseed oil in the diet [38] [39] [46]. According to our funding Lopez-Ferrer et al., [39] also showed that intake of fish oils leads to an impairment of LA metabolism and a deficit of n-6 fatty acids.

In our study, we demonstrated for the first time that *FFAR4*, the main receptor of EPA and DHA [47], was expressed in the peripheral tissues and its expression in the liver and muscles of broiler breeder hen can be regulated by the diet and fish oil supplementation. We found a decreasing effect of fish oil supplementation onthe mRNA expression of *FFAR4* in liver, as well as an increase of lipogenesis (*FASN*) and glucose transporter (*GLUT8*) associated with an increase of *ADIPOQ* expression. This inhibitory effect of fish oil supplementation on FFAR4 expression in liver is in contrast to data previously reported for the rat colon in response to chronic supplementation of diets with 10% fish oil for a period of seven weeks [48]. However, Cornall and colleagues reported a tissue-specific upregulation of FFAR4 expression in rats, such that high-fat diets increased FFAR4 expression in cardiac muscle and EDL skeletal muscle, but not in liver or soleus skeletal muscle [49]. The mechanism of EPA and DHA needs to be explored. We can speculate that a higher percentage of fish oil in the diet may increase the levels of EPA and DHA in metabolic tissue and consequently affect their metabolism.

In agreement with Maddineni et al. (2005) and Ramachandran et al. (2013), we showed that *ADIPOQ*, as well as its receptors *ADIPOR1* and *APIDOR2*, was expressed in chicken adipose tissue, liver and muscles [50] [19]. Food deprivation is known to decrease *ADIPOQ* expression in adipose tissue and the liver [50] and to decrease expression of *ADIPOQ* receptors in adipose tissue [51]. However, our findings showed an increase in *ADIPOQ* mRNA expression in AT sc of Rt animals and no effect on *ADIPOR1* and *ADIPOR2* in either adipose tissue nor liver. We regard to plasma levels, we confirmed that the Rt diet had no effect on circulating ADIPOQ [50]. In addition, plasma level of the high molecular weight ADIPOQ isoform was inversely correlated to body weight and fat mass [52], while we did not find any correlation between circulating ADIPOQ and fat mass in the present study.

In chickens, NAMPT is mostly recognised as a myokine rather than an adipokine [21]. Herein, we showed its expression in adipose tissue, as well as in muscle and liver tissue, without different between tissues. NAMPT is not differentially expressed in adipose tissue from chickens selected for their high fat mass or in lean chickens [53], and no relationship was found between *NAMPT* mRNA expression and fat mass (data no shown).

Concerning the adipokine RARRES2 in avian species, only one of our previous studies revealed the presence of circulating RARRES2 and it expression in adipose tissue, liver and muscle of turkeys [20]. Hence, for the first time, in this study we demonstrated that RARRES2 was expressed in adipose tissue, liver and muscle of broiler breeder hens. Moreover, consistent with studies in humans [54] and rodents [55], we found that circulating RARRES2 was related to fattening. Interestingly, all of the adipokines studied were correlated with plasma triglycerides, phospholipids and insulin concentrations, even if they were unaffected by the diet and supplementation. Furthermore, it is well known that insulin is able to regulate glucose and fatty acid release. Therefore, we can hypothesise that changes in metabolic factors and adipokine expression may be controlled by insulin.

In summary, food restriction and fish oil supplementation modify the metabolic parameters, as well as the adipokine concentrations, in the plasma and tissues in the broiler breeder hens. In addition, we propose two additional tools (BIA and plasma analysis of adipokines) that can be used routinely by breeders to detect possible disorders of metabolism.

## Supporting information

S1 Fig Description of the *in vivo* protocol. From one to 28 days of age (week 4), 320 female breeder chicks received an *ad libitum* diet (free access to food), called a starting diet. At 28 days of age (week 4), animals were distributed into two groups. The first group (n = 160 animals) received a restricted growing diet and the second group (*ad libitum* group; n = 160 animals) received the same diet on a daily basis, but the amount was 1.7 times greater than in restricted animals. From 63 days (week 9) to 273 days of age (week 39), these two groups were each subdivided into two groups, one with fish oil supplementation and one without fish oil. The four groups were: group RNS (restricted unsupplemented); group ANS (*ad libitum* unsupplemented); group RS (restricted supplemented); group AS (*ad libitum* supplemented). During this period, these four groups of animals received three different diets (growing, before laying and laying diets).
(TIF)

S1 Table Composition of the diet with or without fish oil supplementation (%). Crtl: control without fish oil supplementation, Supp: supplemented with fish oil supplementation.
(DOCX)

S2 Table Proportion of fatty acids in the different diets. (DOCX)

S3 Table Fatty acid composition of the OMG750 supplement. (DOCX)

S4 Table Oligonucleotide primer sequences. (DOCX)

## References

[1] Richards MP, Proszkowiec-Weglarz M. Mechanisms regulating feed intake, energy expenditure, and body weight in poultry. Poult Sci. 2007;86(7):1478–90. . 17575199 10.1093/ps/86.7.1478

[2] de Jong IC, van Voorst S, Ehlhardt DA, Blokhuis HJ. Effects of restricted feeding on physiological stress parameters in growing broiler breeders. Br Poult Sci. 2002;43(2):157–68. doi: 10.1080/00071660120121355 . 12047078 10.1080/00071660120121355

[3] Bruggeman V, Onagbesan O, D'Hondt E, Buys N, Safi M, Vanmontfort D, et al Effects of timing and duration of feed restriction during rearing on reproductive characteristics in broiler breeder females. Poult Sci. 1999;78(10):1424–34. . 10536792 10.1093/ps/78.10.1424

[4] Chen LR, Chao CH, Chen CF, Lee YP, Chen YL, Shiue YL. Expression of 25 high egg production related transcripts that identified from hypothalamus and pituitary gland in red-feather Taiwan country chickens. Anim Reprod Sci. 2007;100(1–2):172–85. doi: 10.1016/j.anireprosci.2006.07.005 . 16919900 10.1016/j.anireprosci.2006.07.005

[5] Pan YE, Liu ZC, Chang CJ, Huang YF, Lai CY, Walzem RL, et al Feed restriction ameliorates metabolic dysregulation and improves reproductive performance of meat-type country chickens. Anim Reprod Sci. 2014;151(3–4):229–36. doi: 10.1016/j.anireprosci.2014.10.003 . 25458320 10.1016/j.anireprosci.2014.10.003

[6] M J.A. Broiler breeders: feed restriction and welfare World’s Poultry Science Journal. 2002;58:23–9.

[7] JONG MMVKaICD. Impact of nutrition on welfare aspects of broiler breeder flocks. World's Poultry Science Journal. 2014;70:139–50.

[8] Hocking PM, Maxwell MH, Mitchell MA. Relationships between the degree of food restriction and welfare indices in broiler breeder females. Br Poult Sci. 1996;37(2):263–78. doi: 10.1080/00071669608417858 . 8773836 10.1080/00071669608417858

[9] Robbins KR, McGhee GC, Osei P, Beauchene RE. Effect of feed restriction on growth, body composition, and egg production of broiler females through 68 weeks of age. Poult Sci. 1986;65(12):2226–31. . 3575213 10.3382/ps.0652226

[10] Joseph NS, Robinson FE, Korver DR, Renema RA. Effect of dietary protein intake during the pullet-to-breeder transition period on early egg weight and production in broiler breeders. Poult Sci. 2000;79(12):1790–6. . 11194042 10.1093/ps/79.12.1790

[11] Koppenol A, Delezie E, Aerts J, Willems E, Wang Y, Franssens L, et al Effect of the ratio of dietary n-3 fatty acids eicosapentaenoic acid and docosahexaenoic acid on broiler breeder performance, egg quality, and yolk fatty acid composition at different breeder ages. Poult Sci. 2014;93(3):564–73. doi: 10.3382/ps.2013-03320 . 24604849 10.3382/ps.2013-03320

[12] Poniedzialek-Czajkowska E, Mierzynski R, Kimber-Trojnar Z, Leszczynska-Gorzelak B, Oleszczuk J. Polyunsaturated fatty acids in pregnancy and metabolic syndrome: a review. Curr Pharm Biotechnol. 2014;15(1):84–99. . 24720591 10.2174/1389201015666140330195614

[13] Jones PJH, and S. Kubow. Lipids, sterols, and their metabolites. In: M. E. Shils; MS, A. C. Ross;, B. Caballero; and R. J. Cousins, editor. In Modern nutrition in health and disease2006. p. 92–122.

[14] Decuypere E, Kuhn ER. Alterations in thyroid hormone physiology induced by temperature and feeding in newly hatched chickens. Acta Physiol Pol. 1988;39(5–6):380–94. . 3257053

[15] Kita K, Nagao K, Taneda N, Inagaki Y, Hirano K, Shibata T, et al Insulin-like growth factor binding protein-2 gene expression can be regulated by diet manipulation in several tissues of young chickens. J Nutr. 2002;132(2):145–51. . 11823570 10.1093/jn/132.2.145

[16] Buyse J, Decuypere E, Darras VM, Vleurick LM, Kuhn ER, Veldhuis JD. Food deprivation and feeding of broiler chickens is associated with rapid and interdependent changes in the somatotrophic and thyrotrophic axes. Br Poult Sci. 2000;41(1):107–16. doi: 10.1080/00071660086493 . 10821532 10.1080/00071660086493

[17] Seroussi E, Cinnamon Y, Yosefi S, Genin O, Smith JG, Rafati N, et al Identification of the Long-Sought Leptin in Chicken and Duck: Expression Pattern of the Highly GC-Rich Avian leptin Fits an Autocrine/Paracrine Rather Than Endocrine Function. Endocrinology. 2016;157(2):737–51. doi: 10.1210/en.2015-1634 . 26587783 10.1210/en.2015-1634

[18] Friedman-Einat M, Boswell T, Horev G, Girishvarma G, Dunn IC, Talbot RT, et al The chicken leptin gene: has it been cloned? Gen Comp Endocrinol. 1999;115(3):354–63. doi: 10.1006/gcen.1999.7322 . 10480986 10.1006/gcen.1999.7322

[19] Ramachandran R, Maddineni S, Ocon-Grove O, Hendricks G 3rd, Vasilatos-Younken R, Hadley JA. Expression of adiponectin and its receptors in avian species. Gen Comp Endocrinol. 2013;190:88–95. doi: 10.1016/j.ygcen.2013.05.004 . 23707376 10.1016/j.ygcen.2013.05.004

[20] Diot M, Reverchon M, Rame C, Froment P, Brillard JP, Briere S, et al Expression of adiponectin, chemerin and visfatin in plasma and different tissues during a laying season in turkeys. Reprod Biol Endocrinol. 2015;13:81 doi: 10.1186/s12958-015-0081-5 ; PubMed Central PMCID: PMCPMC4521348. 26228641 10.1186/s12958-015-0081-5 PMC4521348

[21] Krzysik-Walker SM, Ocon-Grove OM, Maddineni SR, Hendricks GL 3rd, Ramachandran R. Is visfatin an adipokine or myokine? Evidence for greater visfatin expression in skeletal muscle than visceral fat in chickens. Endocrinology. 2008;149(4):1543–50. doi: 10.1210/en.2007-1301 . 18096661 10.1210/en.2007-1301

[22] Li J, Meng F, Song C, Wang Y, Leung FC. Characterization of chicken visfatin gene: cDNA cloning, tissue distribution, and promoter analysis. Poult Sci. 2012;91(11):2885–94. doi: 10.3382/ps.2012-02315 . 23091147 10.3382/ps.2012-02315

[23] Vu JP, Larauche M, Flores M, Luong L, Norris J, Oh S, et al Regulation of Appetite, Body Composition, and Metabolic Hormones by Vasoactive Intestinal Polypeptide (VIP). J Mol Neurosci. 2015;56(2):377–87. doi: 10.1007/s12031-015-0556-z ; PubMed Central PMCID: PMCPMC4458420. 25904310 10.1007/s12031-015-0556-z PMC4458420

[24] Yan J, Gan L, Chen D, Sun C. Adiponectin impairs chicken preadipocytes differentiation through p38 MAPK/ATF-2 and TOR/p70 S6 kinase pathways. PLoS One. 2013;8(10):e77716 doi: 10.1371/journal.pone.0077716 ; PubMed Central PMCID: PMCPMC3806819. 24194895 10.1371/journal.pone.0077716 PMC3806819

[25] Yan J, Gan L, Qi R, Sun C. Adiponectin decreases lipids deposition by p38 MAPK/ATF2 signaling pathway in muscle of broilers. Mol Biol Rep. 2013;40(12):7017–25. doi: 10.1007/s11033-013-2821-y . 24178343 10.1007/s11033-013-2821-y

[26] Chabrolle C, Tosca L, Crochet S, Tesseraud S, Dupont J. Expression of adiponectin and its receptors (AdipoR1 and AdipoR2) in chicken ovary: potential role in ovarian steroidogenesis. Domest Anim Endocrinol. 2007;33(4):480–7. doi: 10.1016/j.domaniend.2006.08.002 . 17010558 10.1016/j.domaniend.2006.08.002

[27] Sun JM, Richards MP, Rosebrough RW, Ashwell CM, McMurtry JP, Coon CN. The relationship of body composition, feed intake, and metabolic hormones for broiler breeder females. Poult Sci. 2006;85(7):1173–84. . 16830857 10.1093/ps/85.7.1173

[28] Flachs P, Mohamed-Ali V, Horakova O, Rossmeisl M, Hosseinzadeh-Attar MJ, Hensler M, et al Polyunsaturated fatty acids of marine origin induce adiponectin in mice fed a high-fat diet. Diabetologia. 2006;49(2):394–7. doi: 10.1007/s00125-005-0053-y . 16397791 10.1007/s00125-005-0053-y

[29] Oster RT, Tishinsky JM, Yuan Z, Robinson LE. Docosahexaenoic acid increases cellular adiponectin mRNA and secreted adiponectin protein, as well as PPARgamma mRNA, in 3T3-L1 adipocytes. Appl Physiol Nutr Metab. 2010;35(6):783–9. doi: 10.1139/H10-076 21164549 10.1139/H10-076

[30] Tishinsky JM, Ma DW, Robinson LE. Eicosapentaenoic acid and rosiglitazone increase adiponectin in an additive and PPARgamma-dependent manner in human adipocytes. Obesity (Silver Spring). 2011;19(2):262–8. doi: 10.1038/oby.2010.186 . 20814411 10.1038/oby.2010.186

[31] Lorente-Cebrian S, Bustos M, Marti A, Martinez JA, Moreno-Aliaga MJ. Eicosapentaenoic acid stimulates AMP-activated protein kinase and increases visfatin secretion in cultured murine adipocytes. Clin Sci (Lond). 2009;117(6):243–9. doi: 10.1042/CS20090020 . 19296827 10.1042/CS20090020

[32] Cachaldora P, Garcia-Rebollar P, Alvarez C, De Blas JC, Mendez J. Effect of type and level of fish oil supplementation on yolk fat composition and n-3 fatty acids retention efficiency in laying hens. Br Poult Sci. 2006;47(1):43–9. doi: 10.1080/00071660500475541 . 16546796 10.1080/00071660500475541

[33] Folch J, Lees M, Sloane Stanley GH. A simple method for the isolation and purification of total lipides from animal tissues. J Biol Chem. 1957;226(1):497–509. . 13428781

[34] Carre B, Lessire M, Juin H. Prediction of metabolisable energy value of broiler diets and water excretion from dietary chemical analyses. Animal. 2013;7(8):1246–58. doi: 10.1017/S1751731113000359 . 23527560 10.1017/S1751731113000359

[35] Pearce J. Effects of testosterone on hepatic lipid metabolism in the mature female domestic fowl. J Endocrinol. 1977;75(2):343–4. . 591820 10.1677/joe.0.0750343

[36] Tahmoorespur M, Ghazanfari S, Nobari K. Evaluation of adiponectin gene expression in the abdominal adipose tissue of broiler chickens: feed restriction, dietary energy, and protein influences adiponectin messenger ribonucleic acid expression. Poult Sci. 2010;89(10):2092–100. doi: 10.3382/ps.2010-00772 . 20852099 10.3382/ps.2010-00772

[37] Sell H, Divoux A, Poitou C, Basdevant A, Bouillot JL, Bedossa P, et al Chemerin correlates with markers for fatty liver in morbidly obese patients and strongly decreases after weight loss induced by bariatric surgery. J Clin Endocrinol Metab. 2010;95(6):2892–6. doi: 10.1210/jc.2009-2374 . 20375212 10.1210/jc.2009-2374

[38] Lopez-Ferrer S, Baucells MD, Barroeta AC, Grashorn MA. n-3 enrichment of chicken meat using fish oil: alternative substitution with rapeseed and linseed oils. Poult Sci. 1999;78(3):356–65. . 10090262 10.1093/ps/78.3.356

[39] Lopez-Ferrer S, Baucells MD, Barroeta AC, Grashorn MA. n-3 enrichment of chicken meat. 1. Use of very long-chain fatty acids in chicken diets and their influence on meat quality: fish oil. Poult Sci. 2001;80(6):741–52. . 11441841 10.1093/ps/80.6.741

[40] Matthias Schreiner HWH, Razzazi-Fazeli Ebrahim, Bohm Josef and Moreira Renata G. Effect of different sources of dietary omega-3 fatty acids on general performance and fatty acid profiles of thigh, breast, liver and portal blood of broilers. Journal of the Science of Food and Agriculture. 2005;85:219–26. doi: 10.1002/jsfa.1948

[41] Geier MS, Torok VA, Allison GE, Ophel-Keller K, Gibson RA, Munday C, et al Dietary omega-3 polyunsaturated fatty acid does not influence the intestinal microbial communities of broiler chickens. Poult Sci. 2009;88(11):2399–405. doi: 10.3382/ps.2009-00126 . 19834092 10.3382/ps.2009-00126

[42] Chapkin RS, Kim W, Lupton JR, McMurray DN. Dietary docosahexaenoic and eicosapentaenoic acid: emerging mediators of inflammation. Prostaglandins Leukot Essent Fatty Acids. 2009;81(2–3):187–91. doi: 10.1016/j.plefa.2009.05.010 ; PubMed Central PMCID: PMCPMC2755221. 19502020 10.1016/j.plefa.2009.05.010 PMC2755221

[43] Mori TA, Bao DQ, Burke V, Puddey IB, Watts GF, Beilin LJ. Dietary fish as a major component of a weight-loss diet: effect on serum lipids, glucose, and insulin metabolism in overweight hypertensive subjects. Am J Clin Nutr. 1999;70(5):817–25. . 10539741 10.1093/ajcn/70.5.817

[44] Ruzickova J, Rossmeisl M, Prazak T, Flachs P, Sponarova J, Veck M, et al Omega-3 PUFA of marine origin limit diet-induced obesity in mice by reducing cellularity of adipose tissue. Lipids. 2004;39(12):1177–85. . 15736913 10.1007/s11745-004-1345-9

[45] Calder PC. Omega-3 polyunsaturated fatty acids and inflammatory processes: nutrition or pharmacology? Br J Clin Pharmacol. 2013;75(3):645–62. doi: 10.1111/j.1365-2125.2012.04374.x ; PubMed Central PMCID: PMCPMC3575932. 22765297 10.1111/j.1365-2125.2012.04374.x PMC3575932

[46] Lopez-Ferrer S, Baucells MD, Barroeta AC, Galobart J, Grashorn MA. n-3 enrichment of chicken meat. 2. Use of precursors of long-chain polyunsaturated fatty acids: linseed oil. Poult Sci. 2001;80(6):753–61. . 11441842 10.1093/ps/80.6.753

[47] Oh DY, Talukdar S, Bae EJ, Imamura T, Morinaga H, Fan W, et al GPR120 is an omega-3 fatty acid receptor mediating potent anti-inflammatory and insulin-sensitizing effects. Cell. 2010;142(5):687–98. doi: 10.1016/j.cell.2010.07.041 ; PubMed Central PMCID: PMCPMC2956412. 20813258 10.1016/j.cell.2010.07.041 PMC2956412

[48] Cheshmehkani A, Senatorov IS, Kandi P, Singh M, Britt A, Hayslett R, et al Fish oil and flax seed oil supplemented diets increase FFAR4 expression in the rat colon. Inflamm Res. 2015;64(10):809–15. doi: 10.1007/s00011-015-0864-3 ; PubMed Central PMCID: PMCPMC4565737. 26275932 10.1007/s00011-015-0864-3 PMC4565737

[49] Cornall LM, Mathai ML, Hryciw DH, McAinch AJ. Diet-induced obesity up-regulates the abundance of GPR43 and GPR120 in a tissue specific manner. Cell Physiol Biochem. 2011;28(5):949–58. doi: 10.1159/000335820 . 22178946 10.1159/000335820

[50] Maddineni S, Metzger S, Ocon O, Hendricks G 3rd, Ramachandran R. Adiponectin gene is expressed in multiple tissues in the chicken: food deprivation influences adiponectin messenger ribonucleic acid expression. Endocrinology. 2005;146(10):4250–6. doi: 10.1210/en.2005-0254 . 15976057 10.1210/en.2005-0254

[51] Ghazanfari S. NKaYT. Adiponectin: A novel hormone in birds. Asian Journal of Animal and Veterinary Advances. 2011;6:429–39.

[52] Hendricks GL 3rd, Hadley JA, Krzysik-Walker SM, Prabhu KS, Vasilatos-Younken R, Ramachandran R. Unique profile of chicken adiponectin, a predominantly heavy molecular weight multimer, and relationship to visceral adiposity. Endocrinology. 2009;150(7):3092–100. doi: 10.1210/en.2008-1558 ; PubMed Central PMCID: PMCPMC2703559. 19299452 10.1210/en.2008-1558 PMC2703559

[53] Resnyk CW, Carre W, Wang X, Porter TE, Simon J, Le Bihan-Duval E, et al Transcriptional analysis of abdominal fat in genetically fat and lean chickens reveals adipokines, lipogenic genes and a link between hemostasis and leanness. BMC Genomics. 2013;14:557 doi: 10.1186/1471-2164-14-557 ; PubMed Central PMCID: PMCPMC3765218. 23947536 10.1186/1471-2164-14-557 PMC3765218

[54] Sledzinski T, Korczynska J, Hallmann A, Kaska L, Proczko-Markuszewska M, Stefaniak T, et al The increase of serum chemerin concentration is mainly associated with the increase of body mass index in obese, non-diabetic subjects. J Endocrinol Invest. 2013;36(6):428–34. doi: 10.3275/8770 . 23211604 10.3275/8770

[55] Bozaoglu K, Bolton K, McMillan J, Zimmet P, Jowett J, Collier G, et al Chemerin is a novel adipokine associated with obesity and metabolic syndrome. Endocrinology. 2007;148(10):4687–94. doi: 10.1210/en.2007-0175 . 17640997 10.1210/en.2007-0175

