# Supplementary figures and images for: Effect of different levels of feed restriction and fish oil fatty acid supplementation on fat deposition by using different techniques, plasma levels and mRNA expression of several adipokines in broiler breeder hens
Source: PLoS One. 2018 Jan 24;13(1):e0191121. doi: 10.1371/journal.pone.0191121 (PMC5783386; doi:10.1371/journal.pone.0191121)

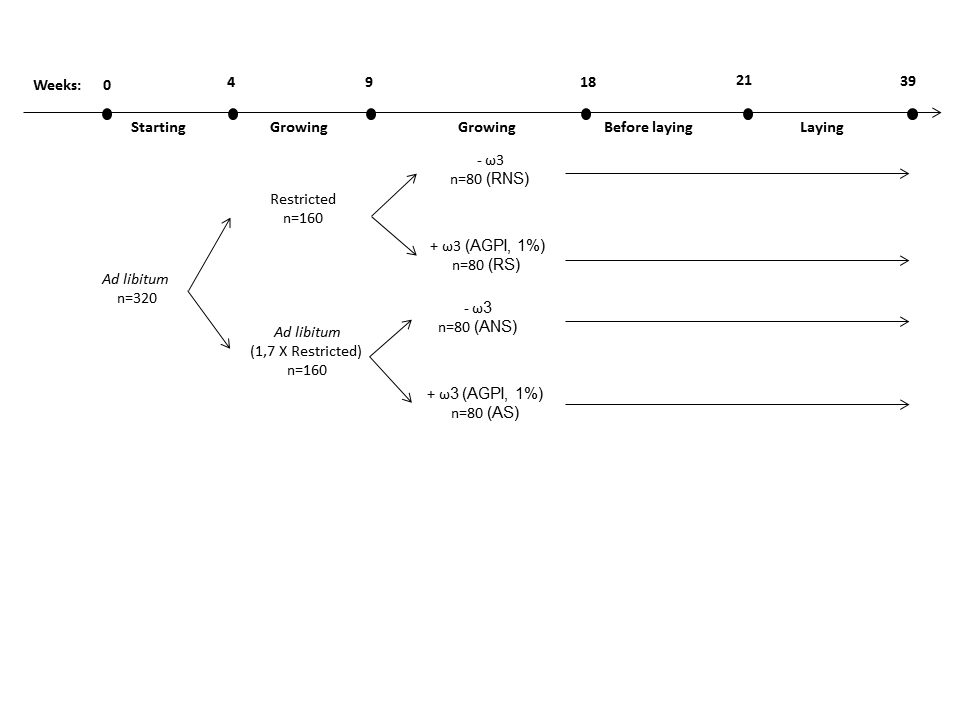

Supplement: S1 Fig — From one to 28 days of age (week 4), 320 female breeder chicks received an ad libitum diet (free access to food), called a starting diet. At 28 days of age (week 4), animals were distributed into two groups. The first group (n = 160 animals) received a restricted growing diet and the second group (ad libitum group; n = 160 animals) received the same diet on a daily basis, but the amount was 1.7 times greater than in restricted animals. From 63 days (week 9) to 273 days of age (week 39), these two groups were each subdivided into two groups, one with fish oil supplementation and one without fish oil. The four groups were: group RNS (restricted unsupplemented); group ANS (ad libitum unsupplemented); group RS (restricted supplemented); group AS (ad libitum supplemented). During this period, these four groups of animals received three different diets (growing, before laying and laying diets). (TIF) [file pone.0191121.s001.tif]
