# Supplementary material for: Effect of different levels of feed restriction and fish oil fatty acid supplementation on fat deposition by using different techniques, plasma levels and mRNA expression of several adipokines in broiler breeder hens
Source: PLoS One. 2018 Jan 24;13(1):e0191121. doi: 10.1371/journal.pone.0191121 (PMC5783386; doi:10.1371/journal.pone.0191121)
Supplement: S1 Table — Crtl: control without fish oil supplementation, Supp: supplemented with fish oil supplementation. (DOCX) [file pone.0191121.s002.docx]

**S1 Table: Composition of the diet with or without ω3 PUFA supplementation (%). Crtl: control without fish oil supplementation, Supp: supplemented with fish oil.**

|  | Starting | Growing | Growing | | Before laying | | Laying | |
| --- | --- | --- | --- | --- | --- | --- | --- | --- |
|  |  |  |  |  |  |  |  |  |
| Weeks | 0 to 4 | 5 to 8 | 9 to 18 | | 19 to 22 | | 23 to 39 | |
|  | Crtl | Crtl | Crtl | Supp | Crtl | Supp | Crtl | Supp |
| Corn | 39.64 | 36.58 | 36.58 | 36.58 | 49.33 | 49.33 | 54.19 | 54.19 |
| Soybean meal (48%) | 29.3 | 3.32 | 3.32 | 3.32 | 14.37 | 14.37 | 13.95 | 13.95 |
| Wheat | 23.86 | 30 | 30 | 30 | 22.22 | 22.22 | 8 | 8 |
| Sunflower oil | 1 | 14 | 14 | 14 | 8 | 8 | 5.8 | 5.8 |
| Soy oil | 2.2 | 1 | 1 | 0 | 1.02 | 0.02 | 2 | 1 |
| Wheat bran | 0 | 11.29 | 11.29 | 11.29 | 0 | 0 | 0 | 0 |
| Sodium bicarbonate | 0.18 | 0.1 | 0.1 | 0.1 | 0.1 | 0.1 | 0.1 | 0.1 |
| Calcium carbonate | 0.86 | 1.08 | 1.08 | 1.08 | 2.1 | 2.1 | 6.6 | 6.6 |
| Phosphate | 2.06 | 1.77 | 1.77 | 1.77 | 2.06 | 2.06 | 2.2 | 2.2 |
| Salt | 0.3 | 0.29 | 0.29 | 0.29 | 0.29 | 0.29 | 0.28 | 0.28 |
| Methionine DL | 0.1 | 0 | 0 | 0 | 0.01 | 0.01 | 0.05 | 0.05 |
| Lysine | 0 | 0.07 | 0.07 | 0.07 | 0 | 0 | 0 | 0 |
| Mineral Premix | 0.5 | 0.5 | 0.5 | 0.5 | 0.5 | 0.5 | 0.5 | 0.5 |
| Soybean | 0 | 0 | 0 | 0 | 0 | 0 | 6.33 | 6.33 |
| OMG 750 | 0 | 0 | 0 | 1 | 0 | 1 | 0 | 1 |
