# Supplementary material for: Effect of different levels of feed restriction and fish oil fatty acid supplementation on fat deposition by using different techniques, plasma levels and mRNA expression of several adipokines in broiler breeder hens
Source: PLoS One. 2018 Jan 24;13(1):e0191121. doi: 10.1371/journal.pone.0191121 (PMC5783386; doi:10.1371/journal.pone.0191121)
Supplement: S2 Table — (DOCX) [file pone.0191121.s003.docx]

**S2 Table: Proportion of fatty acids in the different diets.**

| Diet | Starting | Growing  without OMG | Growing  without OMG | Growing  with OMG | Before laying  without OMG | Before laying  with OMG | During laying  without OMG | During laying  with OMG |
| --- | --- | --- | --- | --- | --- | --- | --- | --- |
| Weeks | 0 to 4 | 5 to 9 | 10 to 18 | 10 to 18 | 19 to 22 | 19 to 22 | 23 to 39 | 23 to39 |
| % of total lipids | 5.2 | 3.44 | 2.99 | 2.68 | 3.11 | 2.67 | 4.94 | 5.18 |
| C18:2 | 52.15 | 53.44 | 54.83 | 49.30 | 53.02 | 44.34 | 52.50 | 49.18 |
| C18:3 | 4.80 | 3.70 | 3.73 | 1.84 | 4.07 | 1.87 | 6.09 | 5.02 |
| C20:4 n-6 | 0.06 | 0.05 | 0.13 | 0.15 | 0.04 | 0.26 | 0.07 | 0.20 |
| C20:5 n-3 | 0.65 | 0.23 | 0.00 | 1.45 | 0.00 | 3.51 | 0.07 | 1.46 |
| C22:4 n-6 | 0.00 | 0.00 | 0.00 | 0.00 | 0.00 | 0.11 | 0.00 | 0.00 |
| C22:5 n-3 | 0.06 | 0.03 | 0.00 | 0.14 | 0.00 | 0.30 | 0.01 | 0.16 |
| C22:6 n-3 | 0.37 | 0.10 | 0.07 | 0.93 | 0.00 | 2.15 | 0.08 | 0.97 |
| n-6 | 52.21 | 53.49 | 54.96 | 49.45 | 53.06 | 44.71 | 52.57 | 49.38 |
| n-3 | 5.88 | 4.06 | 3.80 | 4.36 | 4.07 | 7.83 | 6.25 | 7.61 |
| n-6/n-3 | 8.88 | 13.17 | 14.46 | 11.34 | 13.04 | 5.71 | 8.41 | 6.49 |
