# Supplementary material for: Effect of different levels of feed restriction and fish oil fatty acid supplementation on fat deposition by using different techniques, plasma levels and mRNA expression of several adipokines in broiler breeder hens
Source: PLoS One. 2018 Jan 24;13(1):e0191121. doi: 10.1371/journal.pone.0191121 (PMC5783386; doi:10.1371/journal.pone.0191121)
Supplement: S4 Table — (DOCX) [file pone.0191121.s005.docx]

**S4 Table: Oligonucleotide primer sequences**

| Gene | Product size (bp) | Forward | Reverse |
| --- | --- | --- | --- |
| *EF1-*α | 90 | 5'-AGCAGACTTTGTGACCTTGCC-3' | 5'-TGACATGAGACAGACGGTTGC-3' |
| *ß-ACTIN* | 188 | 5'-ACGGAACCACAGTTTATCATC-3' | 5'-GTCCCAGTCTTCAACTATACC-3' |
| *RPL-15* | 194 | 5'-TGTGATGCGTTTCCTCCTTGG-3' | 5'-CCATAGGTTGCACCTTTTGGG-3' |
| *NAMPT* | 96 | 5'-GCTTCAGCCCATTTGGTGA-3' | 5'-ATCCCGGAACTGGATCTTTTG-3' |
| *RARRES2* | 314 | 5'-CGCGTGGTGAAGGATGTG-3' | 5'-CGACTGCTCCCTAAAGAGGAACT-3' |
| *CMKLR1* | 403 | 5'-CGGTCAACGCCATTTGGT-3' | 5'-GGGTAGGAAGATGTTGAAGGAA-3' |
| *CCRL2* | 391 | 5'-CACGCAGTG TTTGCTTTAAAAGC-3' | 5'-CAACAGCCCACGTGACAATG-3' |
| *ADIPOQ* | 64 | 5'-AATGTCGTGTGCCAACTGGAT-3' | 5'-TTCCAGGCAGCCCATTGT-3' |
| *ADIPOR1* | 350 | 5'-GAATACACACCGAGACGGGC-3' | 5'-GCCCAAGACGCAGACAATGG-3' |
| *ADIPOR2* | 345 | 5'-GAGACTGGCAACATCTGGAC-3' | 5'-TGCGATGCCCAGGACACAAA-3' |
| *FASN* | 74 | 5'-ACCGGTGTT-3' | 5'-TTTCAATGATCCAAATCCAGATA-3' |
| *PPARG* | 66 | 5'-CACTGCAGGAACAGAACAAAGAA-3' | 5'-TCCACAGAGCGAAACTGACATC-3' |
| *FFAR4* | 100 | 5'-TCACCGCCATCCCATTCATC-3' | 5'-GCCACTGAGGGTCATCACATAG-3' |
| *FFAR2* | 386 | 5'-CTCTTTATGGCTGCCCTCAG-3' | 5'-GTAGCCCAGGCTTGGTTGG-3' |
| *FATP1* | 114 | 5'-GCAGCAATCGCAGATCCTAA-3' | 5'-CAACCTGGGGTGAAAGACG-3' |
| *CD36* | 177 | 5'-CTGGGAAGGTTACTGCGATT-3' | 5'-GCGAGGAACTGTGAAACGATA-3' |
| *GLUT8* | 47 | 5'-CGGCAGAGGAGTCCCAGTAC-3' | 5'-GCTTCTTGTTTTGCACTCTCAGGTA-3' |
